# Mathematical analysis of the regulation of competing methyltransferases

**DOI:** 10.1186/s12918-015-0215-6

**Published:** 2015-10-14

**Authors:** Michael C. Reed, Mary V. Gamble, Megan N. Hall, H. Frederik Nijhout

**Affiliations:** Department of Mathematics, Duke University, Durham, 27708 NC USA; Mailman School of Public Health, Columbia University, New YorkNY, 10032 USA; Department of Biology, Durham, 27705 NC USA

**Keywords:** Methyltransferase, Mathematical model, One-carbon metabolism, Regulation

## Abstract

**Background:**

Methyltransferase (MT) reactions, in which methyl groups are attached to substrates, are fundamental to many aspects of cell biology and human physiology. The universal methyl donor for these reactions is S-adenosylmethionine (SAM) and this presents the cell with an important regulatory problem. If the flux along one pathway is changed then the SAM concentration will change affecting all the other MT pathways, so it is difficult for the cell to regulate the pathways independently.

**Methods:**

We created a mathematical model, based on the known biochemistry of the folate and methionine cycles, to study the regulatory mechanisms that enable the cell to overcome this difficulty. Some of the primary mechanisms are long-range allosteric interactions by which substrates in one part of the biochemical network affect the activity of enzymes at distant locations in the network (not distant in the cell). Because of these long-range allosteric interactions, the dynamic behavior of the network is very complicated, and so mathematical modeling is a useful tool for investigating the effects of the regulatory mechanisms and understanding the complicated underlying biochemistry and cell biology.

**Results:**

We study the allosteric binding of 5-methyltetrahydrofolate (5mTHF) to glycine-N-methyltransferase (GNMT) and explain why data in the literature implies that when one molecule binds, GNMT retains half its activity. Using the model, we quantify the effects of different regulatory mechanisms and show how cell processes would be different if the regulatory mechanisms were eliminated. In addition, we use the model to interpret and understand data from studies in the literature. Finally, we explain why a full understanding of how competing MTs are regulated is important for designing intervention strategies to improve human health.

**Conclusions:**

We give strong computational evidence that once bound GNMT retains half its activity. The long-range allosteric interactions enable the cell to regulate the MT reactions somewhat independently. The low *K*_*m*_ values of many MTs also play a role because the reactions then run near saturation and changes in SAM have little effect. Finally, the inhibition of the MTs by the product S-adenosylhomocysteine also stabilizes reaction rates against changes in SAM.

**Electronic supplementary material:**

The online version of this article (doi:10.1186/s12918-015-0215-6) contains supplementary material, which is available to authorized users.

## Background

Methyltransferase reactions, in which methyl groups are attached to substrates, are fundamental to many aspects of cell biology and human physiology. In mammals there are at least 150 methyl transferases that methylate DNA, RNA, lipids, proteins, and small molecules [1, 2]. For example, the methylation of cytosines in DNA is the basis for epigenetic control, the methylation of guanidinoacetate to form creatine is fundamental to energy metabolism, and the methylation of arsenic is an important detoxification pathway. The universal methyl donor in cells is the molecule S-adenosylmethionine (SAM) that is synthesized from the amino acid methionine in the methionine cycle. This poses an unusual but important regulatory challenge for cells. If one methyltransferase (MT) is upregulated by the cell, then there will be a larger flux on that pathway, which should lower the SAM concentration and thereby decrease the flux through the other methylation pathways. That is, it is not clear how the cell can regulate the different methyltransferase pathways independently. Even 40–50 years ago when many methyltransferases were being discovered, researchers understood that this is an important issue and often referred to “competing methyltransferases” [3].

**Fig. 1:**
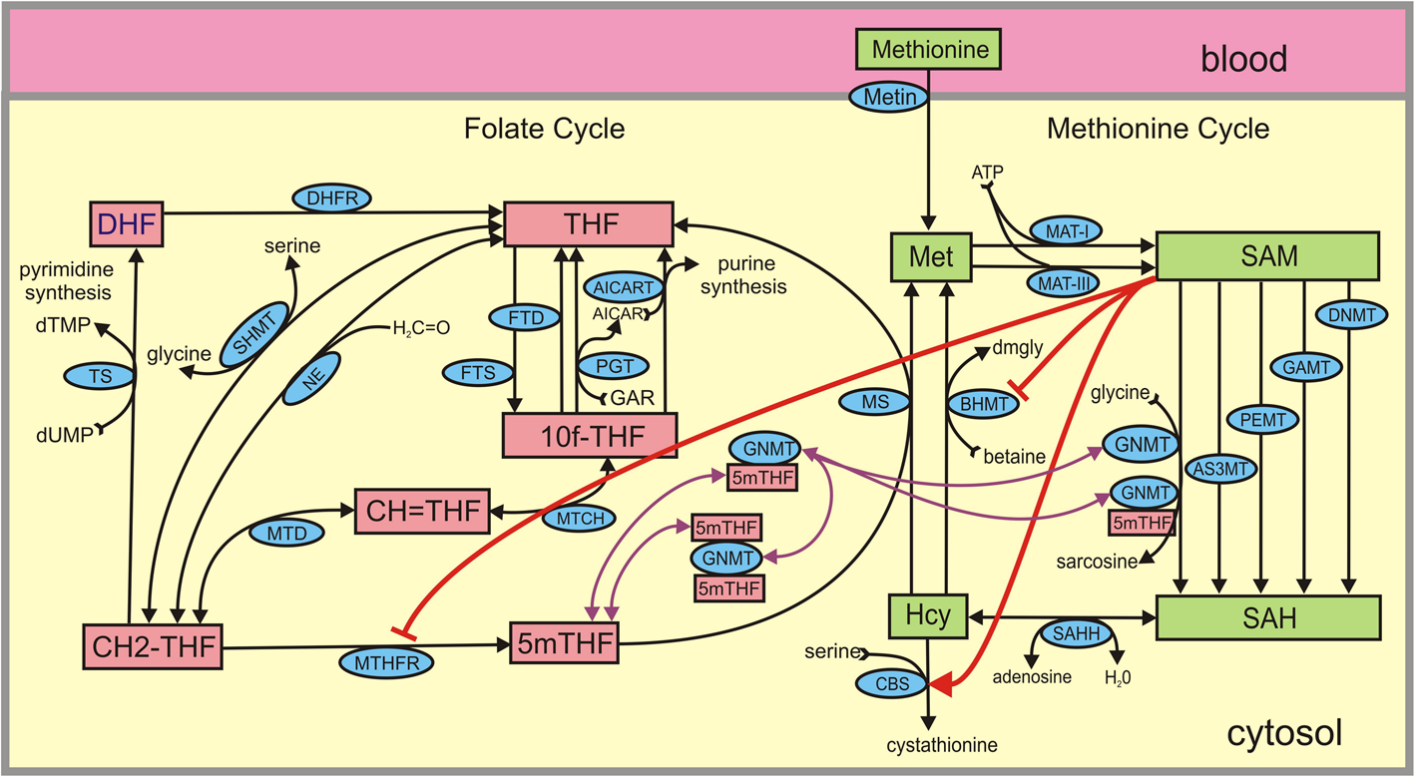
Folate and methionine metabolism with competing methyltransferases. Substrates are indicated by rectangular boxes, green in the methionine cycle and red in the folate cycle, except for GNMT which is both an enzyme and a substrate since it can bind to two molecules of 5mTHF. Each arrow represents a biochemical reaction and the blue ellipse on the arrow contains the acronym of the enzyme that catalyzes the reaction. Substrate abbreviations: Met, methionine; SAM, S-adenosylmethionine; SAH, S-adenosylhomocysteine; Hcy, homocysteine; 5mTHF, 5-methyltetrahydrofolate; THF, tetrahydrofolate; 10fTHF, 10-formyltetrahydrofolate; DHF, dihydrofolate; CH2-THF, 5,10-methylenetrahydrofolate; CH=THF, 5,10-methenyltetrahydrofolate. Enzyme abbreviations: AICAR(T), aminoimidazolecarboxamide ribonucleotide (transferase); FTD, 10-formyltetrahydrofolate dehydrogenase; FTS, 10-formyltetrahydrofolate synthase; MTCH, 5,10-methylenetetrahydrofolate cyclohydrolase; MTD, 5,10-methylenetetrahydrofolate dehydrogenase; MTHFR, 5,10-methylenetetrahydrofolate reductase; TS, thymidylate synthase; SHMT, serine hydroxymethyltransferase; PGT, phosphoribosyl glycinamidetransformalase; DHFR, dihydrofolate reductase; NE, nonenzymatic interconversion of THF and 5,10-CH2-THF; MAT-I,methionine adenosyl transferase I; MAT-III, methionine adenosyl transferase III; GNMT, glycine N-methyltransferase; AS3MT, arsenic methyltransferase; PEMT, phosphotidylethanolamine methyltransferase; GAMT, gunadino-acetate methyltransferase; DNMT, DNA-methyltransferase; SAHH, S-adenosylhomocysteine hydrolase; CBS, cystathionine *β*-synthase; MS, methionine synthase; BHMT, betaine-homocysteine methyltransferase

Many methyl groups used in the MT reactions come directly from dietary input of methionine, betaine, and choline (to make betaine) in the methionine cycle. See Fig. 1. However, the folate cycle can also contribute methyl groups in a process called methylneogenesis. In the folate cycle, a methyl group is removed from serine (making glycine) in the SHMT reaction and is attached to tetrahydrofolate (THF) making methylene-tetrahydrofolate (CH2-THF). See Fig. 1, and its legend for the full names of enzymes. In turn, CH2-THF is reduced by the enzyme MTHFR to 5-methyltetrahydrofolate (5mTHF). In the methionine synthase (MS) reaction, 5mTHF donates the methyl group to homocysteine (Hcy) to make methionine. As we will see, methylneogenesis is partially controlled by the concentration of SAM itself. Mudd and Poole [4] showed that if humans are given diets restricted in methionine, choline, and betaine, the subjects maintained the overall flux through the MT reactions and Davis et al. [5] determined that 100 % of these new methyl groups come from serine. Nijhout et al. [6] showed, using mathematical modeling, that, in addition to dietary input of serine, the mitochondria are an important source of serine in adult cells by converting glycine to serine.

We include five methyltransferases in our model. GNMT, PEMT, and GAMT are included because together they carry most of the methylation flux [7, 8]. DNMT is included because it is an example of the many methyltransferases that have a very low *K*_*m*_ for SAM and it is a methyltransferase that is greatly up regulated when cells divide. Finally, we include AS3MT because the detoxification of arsenic is a special interest of ours. Given the biological importance of the MT reactions, it is not surprising that many regulatory mechanisms have evolved that allow the cell to regulate the methylation fluxes through the different pathways more or less independently. These are: **(1) Long-range interactions** The long-range interactions are depicted by red arrows in Fig. 1. “Long-range” does not refer to distance inside the cell (we assume that all species are at high enough concentration that we can consider the cell to be well-mixed). We use the term “long-range” when a substrate affects an enzyme at a distant location in the network diagram. The concentration of SAM affects the activity of enzymes at distant locations in the biochemical network. SAM activates CBS [9, 10] and inhibits BHMT [11]. As the SAM concentration rises this has the effect of sending more Hcy down the transsulfuration pathway (CBS) and remethylating fewer Hcy (BHMT) to methionine, thus moderating the increase in SAM. In a series of papers, Finkelstein and Martin [12, 13] emphasized that these effects tend to conserve the total mass in the methionine cycle. In addition, SAM inhibits the enzyme MTHFR [14]. Thus when the SAM concentration increases, the concentration of 5mTHF will drop and the MS reaction will go slower, so less Hcy will be remethylated to methionine.**(2) The binding of 5mTHF to GNMT** Zamierowski and Wagner [15] discovered that the GNMT is a major folate binding protein. 5mTHF binds to GNMT and tends to inactivate it; see Fig. 1. Thus, when SAM starts to rise, the inhibition of MTHFR causes the concentration of free 5mTHF to fall causing some of the bound*GNMT-5mTHF* complexes to dissociate. This makes more free GNMT so the GNMT pathway runs faster preventing the SAM concentration from rising too much. Conversely, if SAM starts to fall, the inhibition of MTHFR is partially relieved creating more 5mTHF. The increased 5mTHF binds to more GNMT, lowering the amount of free GNMT available for the GNMT reaction, thus moderating the decline in SAM concentration. Because of these ideas, Wagner and others refer to the GNMT reaction as a “salvage pathway”. When SAM is high many of the excess methyl groups are carried away in the GNMT reaction and when SAM is low the GNMT reaction is constricted to save the methyl groups for the other MT reactions. This view is consistent with the fact that the product of the GNMT reaction, sarcosine, has no known physiological function. It is transported into the mitochondria where the one carbon units are stripped off and (except for one *CO*_2_) returned to the folate cycle in the cytosol.**(3) SAH inhibits the MT reactions** Almost all the MT enzymes are inhibited by S-adenosylhomocysteine (SAH), the universal product of the MT reactions. This also tends to maintain the SAM concentration. If one MT is highly upregulated, the increased flux will raise the concentration of SAH, which will inhibit somewhat more the other MT reactions, thus tending to maintain the concentration of SAM.**(4) Many MT have very low ***K*_*m*_** values for SAM** Some MT enzymes have *K*_*m*_ values for SAM that are of the same magnitude as typical SAM concentrations. This includes GNMT, GAMT, and PEMT, three pathways that carry a major amount of the MT flux. However, many other MT enzymes, for example DNMT, have very low *K*_*m*_ values for SAM [1]. This ensures that even if the SAM concentration fluctuates the velocity of the particular MT reaction won’t change much because it is already on the saturated part of the Michaelis-Menten curve.

The main purpose of this study is to investigate, using mathematical modeling, these regulatory mechanisms. The first step is to use information about the binding of 5mTHF to GNMT recently discovered by Luka and Wagner [16–18] so that we can update and improve our previous models of folate and methionine metabolism in the liver [6, 19, 20]. We present, in Section The binding of 5mTHF to GNMT, evidence from the mathematical modeling that GNMT retains 50 % of its activity when it is once bound by 5mTHF but not when it is twice bound. Then we study the five competing methyltransferases indicated in Fig. 1: GNMT, the salvage pathway, PEMT and GAMT, major MT pathways with moderate *K*_*m*_ values for SAM, DNMT, which has a low *K*_*m*_ value for SAM, and AS3MT, the enzyme in the arsenic detoxification pathway that is a particular interest of ours. In Section Stabilization of methylation reactions by the long-range interactions we show that the long-range interactions moderate the effects on other fluxes when one flux is eliminated or highly upregulated. In Section Stabilization of SAM against changes in methionine input we show how the long-range interactions moderate the effects of changes in methionine input. And, in Section Methylation and folate deficiency we show that even though the SAM concentration depends on total liver folate, the total methylation flux is not markedly affected by folate deficiency. In Section The importance of small *K*_*m*_ values we explain the importance of the small *K*_*m*_ values for SAM of many MTs and in Section The effect of inhibition by SAH we study the inhibition of the MT reactions by SAH. In Section The importance of substrate availability we point out that individual methylation fluxes can be controlled by co-substrate availability as well as enzyme expression level. In Section Arsenic in Bangladesh, we explain why a full understanding of how competing MTs are regulated is important for understanding and designing intervention strategies for human health effects. We also explain that there are times when one would like to break some or all of these homeostatic mechanisms so as to achieve specific changes in the balance of the MT pathways. In addition, we use the model to interpret and understand data from studies in the literature.

## Methods

A schematic diagram of the mathematical model is shown in Fig. 1. The pink boxes indicate the variable substrates in the folate cycle and the green boxes indicate the variable substrates in the methionine cycle. Each arrow represents a biochemical reaction and the blue ellipses on the arrows give the acronyms of the enzymes catalyzing the reactions. Full names of substrates and enzymes are given in the legend to Fig. 1. In addition, the concentration of the free enzyme, GNMT, is treated as a variable in the model, as well as its singly bound and doubly bound forms, *5mTHF-GNMT* and 5*mTHF-GNMT-5mTHF*, respectively. The concentration of each variable satisfies a differential equation that represents mass-balance; that is, the rate of change of the variable (in *μ*M/hr) is simply the sum of the rates of the arrows coming into the variable minus the sum of the rates of the arrows leaving the variable. Thus, the differential equation for [ *SAM*] is

$${} {\fontsize{8.3}{12}{\begin{aligned} \frac{d[\!SAM]}{dt} & = V_{\text{MATI}}([\!Met],[SAM]\!) \; + \; V_{\text{MATIII}}([\!Met],[\!SAM]\!)\\ &\quad -\;V_{\text{GNMT}}([\!SAM],[\!SAH], [\!GNMT], [\!5mTHF\text{-G}NMT]\!) \\ &\quad -\; V_{\text{AS3MT}}([\!SAM],[\!SAH]\!) \; - \; V_{\text{PEMT}}([\!SAM],[\!SAH]\!) \\ &\quad - \; V_{\text{GAMT}}([\!SAM],[\!SAH]\!) \; - \; V_{\text{DNMT}}([\!SAM],[\!SAH]\!) \end{aligned}}} $$

Each *V* is a velocity and the subscript indicates which reaction. Each velocity depends on the current value of the concentrations of the indicated variables. The complicated, usually non-linear, form of this dependence depends on the detailed biochemistry of each of the enzymes. Each of the methylation reactions also depends on the concentration of the substrate being methylated, for example glycine for GNMT and guanadino-acetate for GAMT, though we do not indicate these explicitly. These methylation substrates are taken to be constant in the model because our main interest is the regulation of [ *SAM*] concentration.

The complete mathematical model that consists of 13 differential equations and explicit formulas for each reaction velocity is given in the Additional file 1. Large parts of the model are very similar to the liver folate and methione cycle parts of (larger) models that we have developed in the past to study different aspects of cell metabolism. The contributions of the mitochondria to one-carbon metabolism were studied in [6]. The transulfuration pathway was studied in [21] and whole body folate and methione metabolism was studied in [22]. In the rest of this section we present the new reactions and substrates that we have added in order to study the problem of competing methyltransferases. Full details of the complete model are available in the Additional file 1.

### Binding of 5mTHF to GNMT

In a series of papers, Wagner, Luka, and colleagues have studied the inhibitory effect of 5mTHF on the activity of GNMT [17, 18, 23–25]. GNMT has two binding sites for 5mTHF, so we assume the simple reversible reactions:

$${\fontsize{9}{12}{\begin{aligned} 5mTHF \; + \; GNMT & \rightleftarrows 5mTHF\text{-}GNMT \\ 5mTHF\text{-G}NMT \; + \; 5mTHF &\rightleftarrows 5mTHF\textit{-GNMT-}5mTHF \\[-1pt] \end{aligned}}} $$

with forward and backward rate constants, *k*_1_ and *k*_2_, for the first reaction and *k*_3_ and *k*_4_, for the second reaction. Thus the differential equations for [ 5*mTHF*],[ *GNMT*],[ 5*mTHF-GMNT*],[5*mTHF-GNMT-*5*mTHF*] are:

$${} {\fontsize{8.8}{12}{ \begin{aligned} \frac{d[\!5mTHF]}{dt} & = V_{\text{MTHFR}}([\!CH2 \text{-T}HF],[\!SAM]\!)\\ &\quad- V_{\text{MS}}([\!5mTHF],[\!Hcy]\!)\\ &\quad - k_{1}[\!5mTHF]\!\![\!GNMT]\\ &\quad + k_{2}[\!5mTHF\text{-G}NMT]\\ \frac{d[\!GNMT]}{dt} & = -\, \,k_{1}[\!5mTHF]\!\![\!GNMT] \\ &\quad+ k_{2}[\!5mTHF\text{-G}NMT]\\ \frac{d[\!5mTHF-GNMT]}{dt} & = k_{1}[\!5mTHF]\!\![\!GNMT] \\ &\quad- k_{2}[\!5mTHF\text{G}NMT]\\ &\quad - k_{3}[\!5mTHF\text{-G}NMT]\!\![\!5mTHF] \\ &\quad+ k_{4}[\!5mTHF\textit{-GNMT-}5mTHF]\\ \frac{d[\!15mTHF\text{G}NMT5mTHF]}{dt} & = +\,\,k_{3}[\!5mTHF\textit{-GNMT}]\!\![5mTHF] \\ &\quad- k_{4}[\!5mTHF\textit{-GNMT-}5mTHF] \end{aligned}}} $$

We choose the rate constants *k*_1_=50,*k*_2_=1,*k*_3_=1,*k*_4_=1.6 so that the *K*_*D*_ values are those found in Table two of [17].

### GNMT

The kinetics of GNMT for SAM are cooperative and we take the Hill coefficient to be *n*=2 as suggested in [24] and we use *K*_*m*_=100 *μ*M as indicated in [1]. The inhibition by SAH is competitive [26] and has *K*_*i*_=35 *μ*M [1]. Of course, the reaction has glycine as a substrate but we take the glycine concentration to be constant so it’s effect is included in *V*_*max*_=4000 *μ*/hr. Thus,

$${}\begin{aligned} &V_{\text{\scriptsize GNMT}}([\!SAM],[\!SAH],[\!GNMT], [\!5mTHF\text{-G}NMT] \!) \; \\ &\qquad= \; \frac{V_{max}[\!SAM]^{2}}{\left.\left(K_{m}\left(1 + \frac{[\!SAH] }{K_{i}}\right)\right)^{2} +\, [\!SAM^{2}]\right)}. \end{aligned} $$

where

$${} V_{max} \; = \;(4000)([\!GNMT]\,+\, (.5)[\!5mTHF\text{-G}NMT]\!). $$

This formula for *V*_*max*_ resulted from our *in silico* experiments described in the first section of Results. The concentration of free GNMT, [GNMT], is a variable in our model. GNMT can be bound by one or two molecules of 5mTHF. Our simulations and the data in [17] suggest strongly that once bound GNMT, namely [ 5*mTHF*-G*NMT*], retains 50 % of it’s activity. The factor 4000 is chosen so that the GNMT has a normal reaction velocity comparable to the reaction velocities of PEMT and GAMT, the two other methyl transferases that carry much of the methylation flux.

### AS3MT

Inorganic arsenic is metabolized in two methylation steps catalyzed by AS3MT. The first step uses utilizes a methyl group from SAM and is followed by a reduction step to produce methylarsonic acid (MMA). The second step uses utilizes a methyl group from SAM and is followed by a reduction step to produce dimethyarsinic acid (DMA), which is readily exported from the liver and cleared in the urine. We have recently studied the biochemistry of these methylation steps that are quite complicated [27]. For, example the first step shows substrate inhibition by inorganic arsenic and product inhibition by MMA and glutathione (GSH) both sequesters the arsenic compounds and activates AS3MT. In our study here, we are mainly interested in studying the availability of methyl groups from SAM, so we take the arsenic concentrations and the GSH concentration to be constant, and model just the first methylation step. SAM shows substrate inhibition for AS3MT [24], but the effect is small and occurs only at very high SAM concentrations, so we ignore it. Thus, the velocity of methylation is taken to be:

$${} {\fontsize{9}{12}{\begin{aligned} V_{\text{AS3MT}}\!\left([\!SAM],[SAH]\!\right) \; = \; \frac{V_{max}[\!SAM]}{\left(K_{m}\left(1 + \frac{[SAH]}{K_{i}}\right) +\, [\!SAM]\!\right)}. \end{aligned}}} $$

We take the *K*_*m*_ of AS3MT for SAM to be 50 *μ*M as determined in [28]. It is known that SAH inhibits AS3MT [29, 30], but the nature of the inhibition and the *K*_*i*_ are not known. We’ll assume the inhibition is competitive and take *K*_*i*_=10 *μ*M, which is typical of other methyltransferases. A high, but realistic arsenic load is 1 *μ*M in liver [31] and a typical flux would be the order of magnitude of 1 *μ*M/hr. So, we choose *V*_*max*_=5 *μ*/hr, which accomplishes this given that a typical SAM concentration is 24 *μ*M.

### PEMT

The velocity of the PEMT reaction is given by

$${} V_{\text{PEMT}}([\!SAM],[\!SAH]\!) = \frac{V_{max}[\!SAM]}{(K_{m} \,+\, [\!SAM]\!)\left(1 + \frac{[SAH]}{K_{i}}\!\right)}. $$

The inhibition by SAH is non-competitive [32]. We choose *K*_*m*_=18.2 *μ*M for SAM and *K*_*i*_=3.8 *μ*M for SAH as indicated in [1]. The reaction depends on phosphatidylethanolamine but since we take its concentration to be constant we fold that dependence into the *V*_*max*_. The value *V*_*max*_=250 *μ*M/hr was chosen so that the flux of the PEMT reaction is comparable to the fluxes of the GNMT and GAMT reactions, the two other methyl transferases that carry much of the methylation flux.

### GAMT

The velocity of the GAMT reaction is given by

$${} V_{\text{\scriptsize GAMT}}\left([\!SAM],[SAH]\!\right) \; = \; \frac{V_{max}[SAM]}{K_{m}\left(1 + \frac{[SAH]}{K_{i}}\right) +\, [\!SAM]}. $$

The inhibition by SAH is competitive [33, 34]. We choose *K*_*m*_=49 *μ*M for SAM and *K*_*i*_=16 *μ*M for SAH as indicated in [1]. The reaction depends on the effective concentration of the cytosine substrates, but since we take its concentration to be constant we fold that dependence into the *V*_*max*_. The value *V*_*max*_=100 *μ*M/hr was chosen so that the flux of the GAMT reaction is comparable to the fluxes of the GNMT and PEMT reactions, the two other methyl transferases that carry much of the methylation flux.

### DNMT

The velocity of the DNMT reaction is given by

$${} V_{\text{GAMT}}([\!SAM],[\!SAH]\!) \; = \; \frac{V_{max}[\!SAM]}{K_{m}\left(1 + \frac{[SAH]}{K_{i}}\right) +\, [\!SAM]}. $$

The inhibition by SAH is competitive [35]. We choose *K*_*m*_=1.4 *μ*M for SAM and *K*_*i*_=1.4 *μ*M for SAH as indicated in [36]. The reaction depends on guanidinoacetate but since we take its concentration to be constant we fold that dependence into the *V*_*max*_. The value *V*_*max*_=12.5 *μ*M/hr was chosen so that the flux of the DNMT reaction is normally (when the cell is not dividing) much less than the fluxes of GNMT, PEMT, and GAMT.

The fluxes of each of the MT reactions depends on the availability of co-substrates, for example phosphatidylethanolamine for the PEMT reaction or guanidinoacetate (*gaa*) for the GAMT reaction. We take these co-substrates to be constant in our simulations, so, for simplicity, the co-substrate terms are not shown in the above formulas. Full formulas with the co-substrates in the cases of AS3MT, GNMT, GAMT, and PEMT are given in the Additional file 1. The *V*_*max*_ values given here and in the Additional file 1 differ somewhat because of the presence of these extra terms.

### On the use of Michaelis-Menten kinetics

Almost all of our formulas for reactions velocities have Michaelis-Menten form, for example *V*=*V*_*max*_[ *S*]/(*K*_*m*_ + [ *S*]), modified to include the effects of product inhibition, substrate inhibition, or long-range allosteric interactions. All of the explicit formulas are in the Additional file 1. These formulas are simplifications of underlying biochemical processes that are more complicated. For example, the actual sequence of steps of the MS reaction, worked out by Rowena Matthews and others, is very long and complicated (and involves SAM). But we are mainly interested in how overall reaction velocity depends on the concentrations of Hcy and 5mTHF, so we use the simple bi-molecular Michaelis-Menten formula until we think that the details of the mechanism are important for the questions we are trying to answer. Deciding what level of biological and biochemical detail to include is always a difficult and important issue. We use simple formulations until the data that we are trying to understand suggests to us that the details on a lower level may be relevant.

## Results and discussion

### The binding of 5mTHF to GNMT

It was established as early as 1985 by Wagner and co-workers [23] that 5mTHF inhibits the enzyme GNMT; 15 % inhibition was shown at 0.1 *μ*M of 5mTHF pentaglutamate, 50 % inhibition at 1 *μ*M and 90 % inhibition at 10 *μ*M. Since then, Wagner, Luka, and co-workers have investigated the structure of GNMT and the binding of 5mTHF. In [24] it was shown that the binding of SAM to GNMT is cooperative and depends on the 5mTHF concentration and GNMT and shows substrate inhibition for SAM at high SAM concentrations. In [16, 37] the structure of GNMT was elucidated and it was shown that GNMT can bind two molecules of 5mTHF. In [17], KD values for the binding of 5mTHF to GNMT are given and inhibition curves and free versus bound 5mTHF curves are given.

**Fig. 2:**
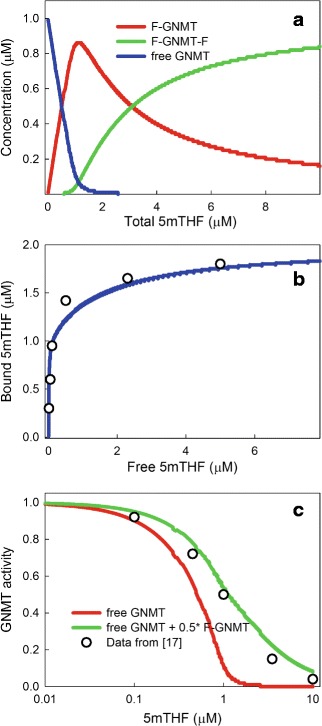
Binding of 5mTHF to GNMT. Panel **a** shows the concentrations of the three substrates, free *GNMT*(blue curve), *G* *N* *M* *T*-5*m* *T* *H* *F*(red), and 5*m* *T* *H* *F*-*G* *N* *M* *T*-5*m* *T* *H* *F*(green) as a function of the concentration of 5*m* *T* *H* *F*. Total *GNMT* concentration is 1 *μ*M. Panel **b** shows the concentration of bound 5*m* *T* *H* *F* as a function of total 5*m* *T* *H* *F*. The open circles are data replotted from [17], Fig. 2, and the blue curve is the model calculation. The concentration of *GNMT* is 1 *μ*M. Since the concentration of bound 5*m* *T* *H* *F* goes well above 1 *μ*M, this proves conclusively that two 5mTHF can bind to each *G* *N* *M* *T*. The data (open circles) in Panel **c**, taken from [17], Fig. 3, shows how the activity of GNMT decreases from 1 (normal) as the concentration of 5mTHF increases. The red curve shows the activity of GNMT, as computed by the model, if one assumes that once and twice bound GNMT lose all activity. The green curve shows the activity of GNMT as computed by the model if one assumes that once and GNMT has 50 % activity and twice bound GNMT has no activity. The green curve gives an excellent fit to the data

Left open was the question of what the activity of the enzyme GNMT is when it is once bound or twice bound with 5mTHF. To investigate this question we used the four differential equations given in Methods for [ 5*mTHF*],[ *GNMT*],[ 5*mTHF*-*GNMT*] and [ 5*mTHF*-*GNMT*-5*mTHF*]. GNMT is 1–3 % of liver protein [26] and in [37] the GNMT concentration in liver was measured to be 1 *μ*M, so we assume that GNMT concentration in our *in silico* experiments and take the KD values from [17]. Our results are shown in Fig. 2. Panel a shows the concentrations of [ *GNMT*],[ 5*mTHF*-*GNMT*] and [ 5*mTHF*-*GNMT*-5*mTHF*] as a function of the total concentration of [ 5*mTHF*] in the medium. Panel b shows our computed model curve of bound 5mTHF versus free 5mTHF along with the data points from Figure 2 of [17]. The match of the computed curve to the data is very good. Note that since there is 1 *μ*M of GNMT in the mixture and the curve and the data rise to 1.7 *μ*M, this shows conclusively that two molecules of 5mTHF can bind to each GNMT. Finally, in Panel c we show the data points from the inhibition curve given in Figure 3 of [17] and two different model curves. The red curve shows the % activity of GNMT assuming that free GNMT has 100 % activity and once bound and twice bound GNMT have 0 % activity. The curve is nowhere near the data. On the other hand, the green curve shows the computed % activity of GNMT assuming that free GNMT has 100 % activity, once bound GNMT has 50 % activity, and twice bound GNMT has 0 % activity. The computed curve lies very close to the data points. We conclude from these computational experiments that it is very likely that once bound GNMT retains 50 % of its activity.

**Fig. 3:**
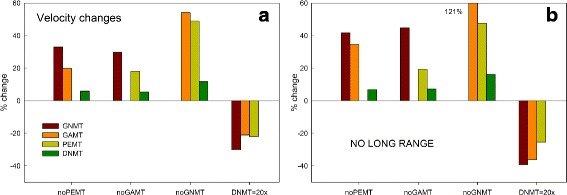
Steady state changes in methylation velocities when one velocity is knocked out or upregulated. Panel **a** shows the percentage changes in methylation velocities in the model when either PEMT, GAMT, or GNMT is knocked out, or when DNMT is 20-fold upregulated. Because of the long-range interactions, GNMT flux changes a lot so that the change is SAM is modest and the PEMT and GAMT fluxes do not change so much. Panel **b** shows the results of the same experiments in the case that the long-range interactions are turned off. All the changes are greater than those in Panel **a**

### Stabilization of methylation reactions by the long-range interactions

We performed various computational experiments that show the effects of the long-range interactions. Since our results are presented as percentage change from normal, we begin by briefly discussing the normal steady state concentrations and velocities in the model that are indicated in Table 1. Our mathematical model, shown schematically in Fig. 1, differs from previous models [19, 20, 22] in three ways: (1) We have added the PEMT, GAMT, and AS3MT methylation reactions; (2) We treat the binding of 5mTHF to GNMT in much more detail; (3) We have lowered the methionine input to the liver to 50 *μ*M/hr so that the steady state concentration of SAM (23.97) is in the range indicated for humans in [38, 39]. The methionine concentration is correspondingly low. Total folate concentration in the liver is 20 *μ*M [19] and the 5mTHF concentration is 5.35 *μ*M [19]. The fraction of the flux around the methionine cycle that enters the transsulfuration pathway (“frac”) is 0.5 [12].

**Table 1:** Normal values of concentrations (*μ*M) and velocities (*μ*M/hr)

Concentrations		Velocities	
Met	17.97	metin	50
SAM	23.97	VAH	100.25
SAH	3.89	VGNMT	43.97
Hcy	2.27	VDNMT	2.05
5mTHF	5.35	VGAMT	25.42
Folate total	20	VPEMT	27.53
Frac	.50	VASMT	1.28
		VMS	23.65
		VBHMT	26.60

The flux around the methionine cycle, 100.25 *μ*M/hr, is given by the net flux of the reaction from SAH to Hcy. The three major methylation fluxes are those catalyzed by GNMT, PEMT, and GAMT [7, 8] and they carry most of the normal flux in our model. The fluxes through DNMT and AS3MT are small representing two of the many other methyltransferases. The remethylation flux from Hcy to Met in the liver is almost equally divided between MS and BHMT and the CBS flux at steady state must be 50 *μ*M/hr since that is the methionine input.

Our first computational experiments investigate how sudden drastic changes in the amounts of PEMT, GAMT, GNMT, or DNMT affect the other fluxes. In Panel a of Fig. 3, we show the percentage change of each of the other fluxes if one flux is set to zero or if the amount of DNMT is multiplied by a factor of 20. Note that a flux could become zero if either the enzyme expression level goes to zero or the co-substrate concentration is zero. Across the bottom we indicate which of the fluxes has been altered. As expected, if one enzyme is set to zero the other fluxes go up and if DNMT is upregulated by a factor of 20, the other fluxes go down. The percentage changes when PEMT or GAMT is set to zero (appoximately 20 %) are consistent with the PEMT and GAMT knockout changes discussed in [7]. The most dramatic changes come when GNMT is knocked out, which was also seen in [7]. When DNMT is upregulated by a factor of 20, the three fluxes of GNMT, PEMT, and GAMT decrease modestly. One can clearly see the influence of GNMT as the “salvage” pathway first described by Wagner, Briggs, and Cook [23]. If PEMT is knocked out GNMT flux goes up a lot so that the GAMT flux does not rise very much. Similarly, when DNMT is upregulated, the GNMT flux drops dramatically and so PEMT and GAMT do not drop as much.

Panel b of Fig. 3 shows what happens if we do the same experiments but turn off the long-range interactions. In every case the changes to the fluxes are larger when one flux is knocked out or (in the case of DNMT) upregulated. Some differences are quite large. If GNMT is knocked out, then GAMT increases by 55 % in the presence of the long-range interactions and increases by 121 % with no long-range interactions.

**Fig. 4:**
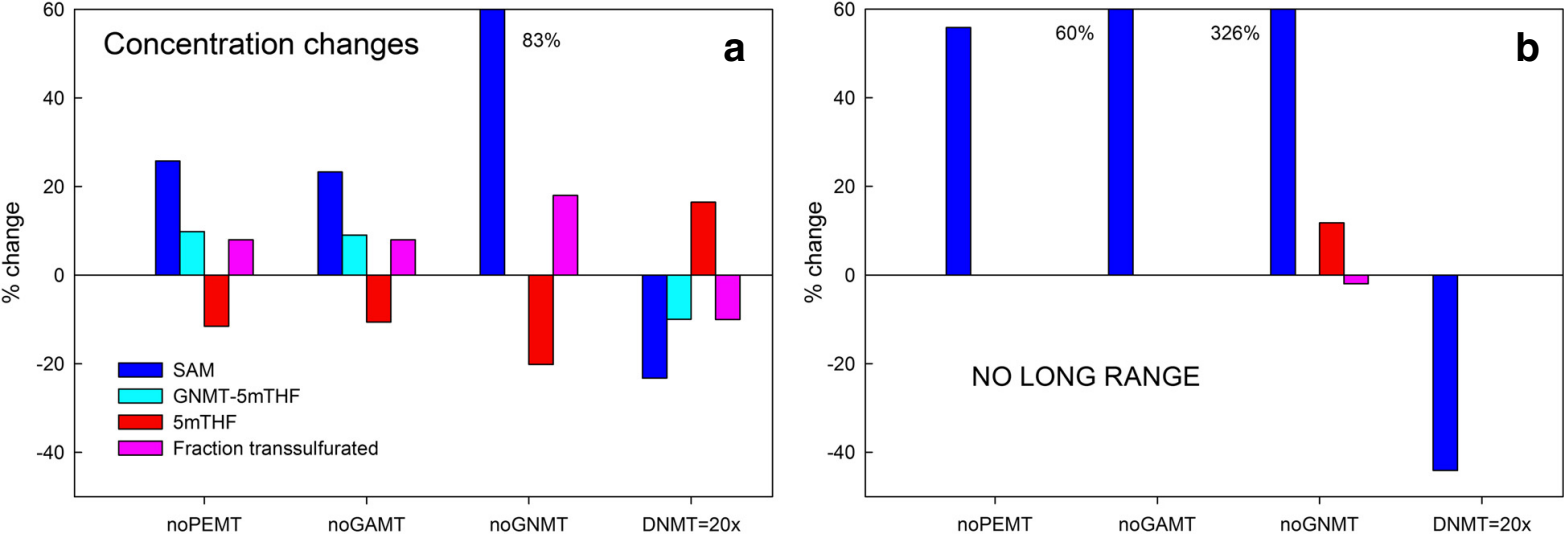
Steady state changes in substrate concentration and fraction transsulfurated when one velocity is knocked out or upregulated. Panel **a** shows the percentage changes in SAM, GNMT-5mTHF, and 5mTHF concentrations, and fraction transsulfurated in the model when either PEMT, GAMT, or GNMT is knocked out, or when DNMT is 20-fold upregulated. The long-range interactions cause substantial changes in all four variables and these changes are what make the velocity changes in Panel a of Fig. 3 relatively modest. Panel **b** shows what happens when the long-range interactions are turned off. The GNMT-5mTHF, 5mTHF concentrations, and fraction transsulfurated don’t change at all (except in the case of GNMT knockout), but SAM undergoes enormous changes

By examining Fig. 1 and Panel a of Fig. 4, we can see how these long-range interactions work. Consider the case where PEMT is knocked out. SAM goes up 25 % inhibiting MTHFR more and thus lowering 5mTHF by 14 %. This drives the reaction of 5mTHF with GNMT towards dissociation so there is less double bound GNMT and more single bound GNMT (by about 10 %), which increases the flux in the GNMT reaction. This is why (see Fig. 3, Panel a) the GNMT flux goes up a lot, but the GAMT flux goes up less (about 20 %) and DNMT flux goes up very little. Finally, the fraction of flux transsulfurated goes up, because increased SAM stimulates CBS and inhibits BHMT, and this moderates the increase in SAM. The analogous scenario happens when GAMT is knocked out.

The opposite scenario happens if DNMT is upregulated by a factor of 20. The steady state value of SAM goes down by 22 %, which removes inhibition from MTHFR, so 5mTHF goes up by 18 %. This drives the reaction of 5mTHF with GNMT towards binding and so the amount of once bound GNMT goes down. Thus less flux goes through the GNMT reaction allowing the the PEMT and GAMT reactions to decrease more modestly (Fig. 3, Panel a). Finally, since SAM goes down, the fraction transulfurated decreases by 10 %, which recycles more methyl groups back to methionine for the transmethylation reactions.

This beautiful mechanism is broken if GNMT is knocked out. As one can see in Panel a of Fig. 4, SAM increases dramatically and this causes very large increases in the fluxes through PEMT, GAMT, and DNMT (Panel a of Fig. 3). And if there are no long-range interactions at all (Panel b of Fig. 4), SAM goes way up or way down without changing GNMT-5mTHF, 5mTHF, or the fraction transsulfurated much. The large changes in SAM drive the increased changes in the fluxes seen in Panel b of Fig. 3.

### Stabilization of SAM against changes in methionine input

**Fig. 5:**
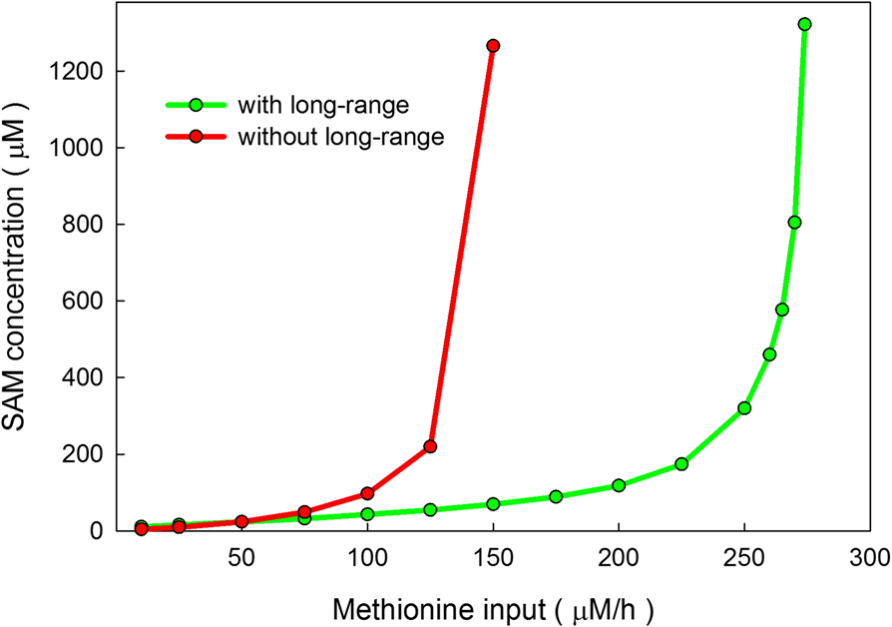
Dependence of SAM on methionine input. When the long-range interactions are present (green curve), the steady state value of SAM increases slowly and linearly as methionine input is increased over a wide range. When the long-range interactions are removed (red curve), the steady state value of SAM increases more rapidly and accelerates when methionine input becomes high. Both curves show the “substrate switch” [41] in which the steady state value of SAM rises modestly at first as methionine input rises but after a certain point rises extremely rapidly

Since SAM is the universal methyl group donor, its concentration determines the availability of methyl groups for the transmethylation reactions. This presents a problem for the cell since the precursor of SAM is methionine and the methionine concentration in the blood varies dramatically during the day due to meals. We have previously carried out a fluctuation analysis that showed how the long-range interactions greatly dampen the fluctuations in methionine input and cause the flux through the DNMT reaction to remain quite stable [20]. Here we show how the steady state value of SAM depends on methionine input (Fig. 5). With the long-range interactions present (green curve) the steady state value of SAM increases fairly slowly and linearly as methionine input increases from 10 *μ*M/hr to 100 *μ*M/hr. When the long-range interactions are removed (red curve), the steady state value of SAM increases much more rapidly and accelerates as the methionine input increases. In both cases the SAM concentration increases dramatically at high values of methionine input. This is the behavior seen in the models of [40, 41] where they call it a “substrate switch,” and the *in vitro* experiments [41]. Figure 5 shows that the switch occurs much earlier without the long-range interactions. These results are consistent with the classic experiments of Finkelstein and Martin who fed rats diets with different amounts of methionine and measured liver SAM (Table 1 in [42]). As the methionine content of their diets increased, rats showed relatively modest increases in the SAM concentrations in their livers until at very high methionine diets SAM increased dramatically. All homeostatic mechanisms, including the long-range interactions discussed here, can be broken if inputs become too extreme, a phenomenon that is discussed further in [43].

### Methylation and folate deficiency

**Fig. 6:**
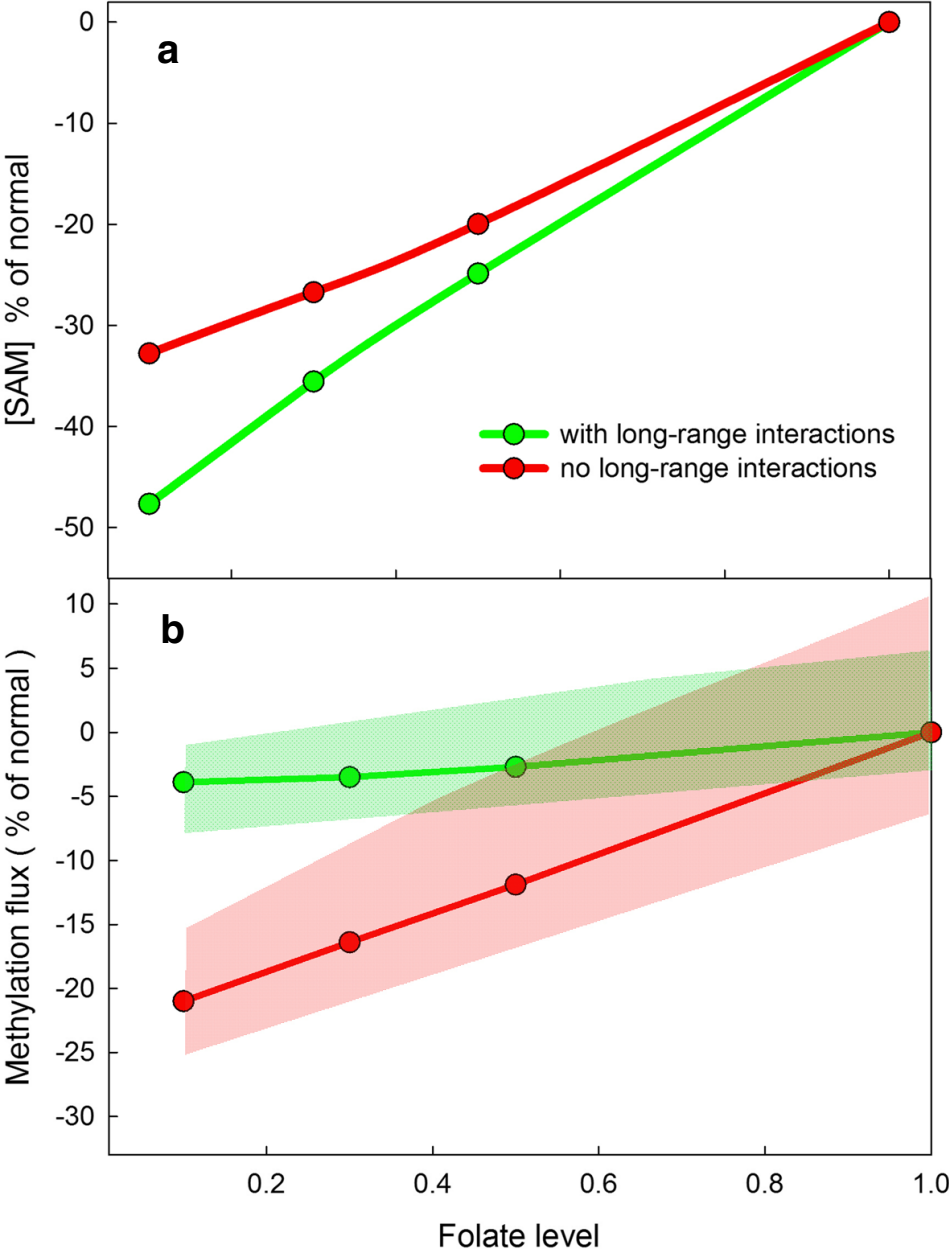
The effect of folate deficiency on total methylation. The curves in Panel **a** show that the SAM level is a linear function of total folate level in the liver whether the long-range interactions are present or not. This is consistent with previous studies (see text). Panel **b** shows that the total methylation rate (green curve) is remarkably stable as the level of total folate decreases, despite the drop in SAM, if the long-range interactions are present. If the long-range interactions are absent (red curve), the rate of total methylation decreases rapidly as total liver folate decreases. In order to show dependence on parameters, we conducted the following *in silico* experiments. In each experiment we multiplied the *V*_*max*_ of each of the 11 *V*_*max*_ values in the methionine cycle by a number drawn randomly and independently from the interval [0.9,1.1]. We then generated the green and red curves corresponding to those parameters. The shaded green and red areas show the envelopes of those curves. Although the curves depend on the choices of parameters, the main conclusion remains the same: the long-range interactions protect total methylation flux against folate deficiency

There is a positive statistical association between liver folate status and liver SAM concentration [44]. Using a mathematical model of folate and methionine metabolism, we showed that over normal physiological ranges liver SAM concentration is a linear increasing function of total liver folate [45] and later more detailed models show the same behavior [21]. Figure 6, Panel a, shows that this is true both in the presence of and without the long-range inhibitions. The reason is easy to understand. Higher total folate means higher 5mTHF, which drives the MS reaction faster and remethylates more Hcy to become Met. Much more interesting is the dependence of total methylation rate (i.e., the sum of all five fluxes in the model) on folate status as shown in Panel b of Fig. 6. In the presence of the long-range interactions, there is almost no decrease in total methylation flux (despite the decrease in SAM) as folate deficiency becomes more and more severe (green curve). In contrast, total methylation flux goes down rapidly as folate deficiency increases if the long-range interactions are absent (red curve). Thus, one evolutionary purpose of the long-range interactions may have been to protect the methylation reactions against the folate deficiencies that surely developed during Fall and Winter when green vegetables were scarce.

For all of our model simulations it is natural to ask how sensitive the results are to the exact choices of parameters. The answer is not much. There are 11 *V*_*max*_ values for the velocities around the methionine cycle. We conducted experiments in which each these *V*_*max*_ values was modified by multiplying it by a number chosen randomly between 0.9 and 1.1 (maximal 10 % variation). All the *V*_*max*_ values were changed simultaneously and independently. The green and red shaded areas in Fig. 6b are the envelopes of the values we obtained in 10 such simulations. As one can see, the fundamental conclusion of Fig. 6b remains the same, whichever set of *V*_*max*_ values one has chosen: the long-range interactions stabilize total methylation rate against folate deficiency. The individual variation of enzyme parameters like *V*_*max*_ and *K*_*m*_ values is not known. We chose 10 % not to represent real biological variation, but simply to show that the model results and conclusions do not depend sensitively on the exact choices of parameters.

### The importance of small *K*_*m*_ values

**Fig. 7:**
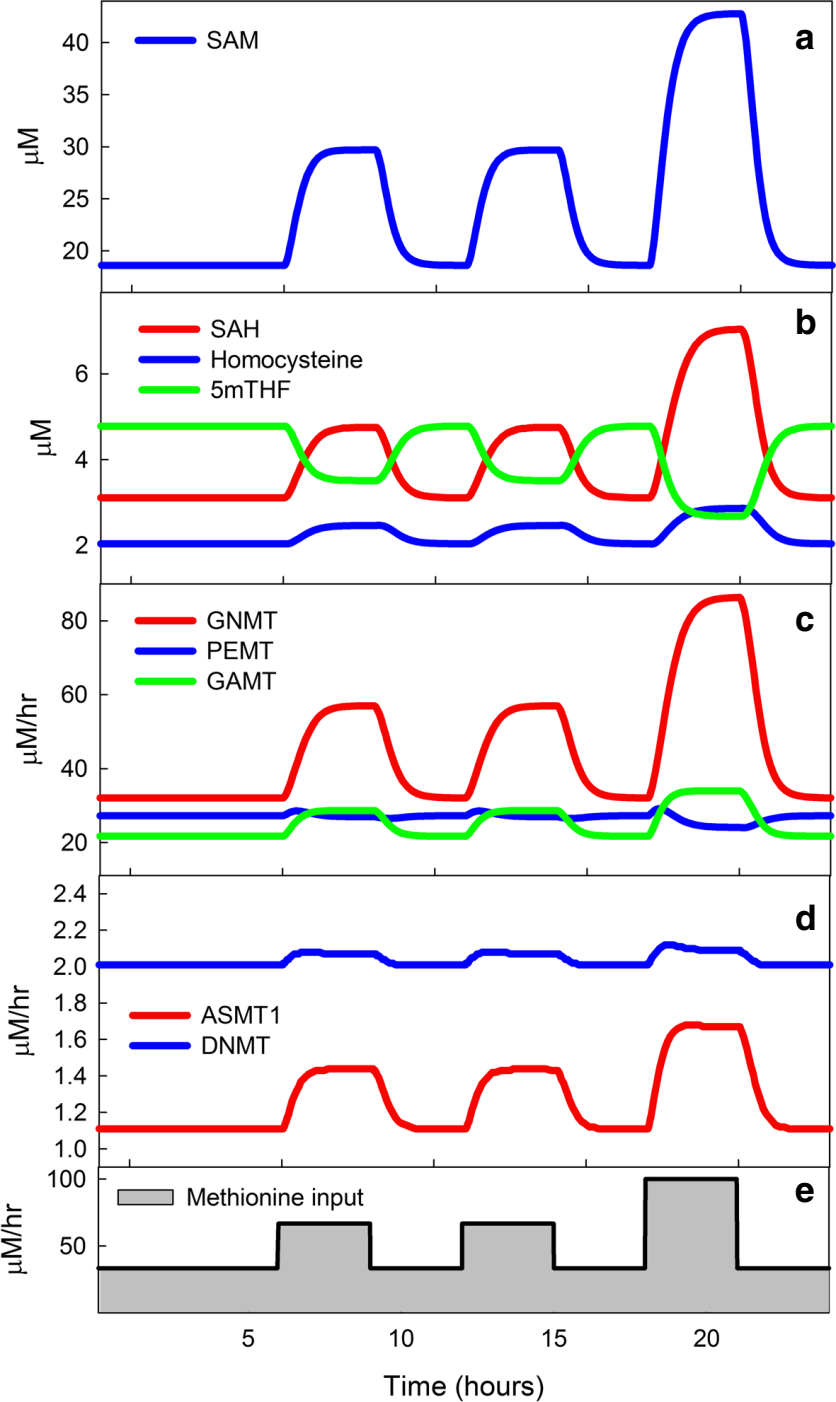
The effect of meals on competing methyltransferases. The input of methionine into the liver Panel **e** was varied during a 24 hour period: 33.33 *μ*M/hr until breakfast, then 66.67 *μ*M/hr for 3 hours, similarly for lunch, and for 3 hours after dinner the input is 100 *μ*M/hr. Panel **a** shows the large deviations in SAM due to the methionine input changes. Panel **b** shows that SAH and Hcy track the changes in SAM, but 5mTHF has the opposite changes because SAM inhibits MTHFR (see Fig. 1). The changes in Hcy are small because SAM stimulates CBS. Panel **c** shows the time course of the fluxes through GNMT, PEMT, and GAMT. As explained in the text, GNMT goes up rapidly as SAM increases taking most of the extra methyl groups so that the changes to GAMT and PEMT are modest. The changes to PEMT are exceptionally small (see the text for Section The effect of inhibition by SAH). Panel **d** shows the fluxes through AS3MT and DNMT. The changes to DNMT are exceptionally small (see the text for Section The importance of small *K*_*m*_ values) because the *K*_*m*_ of DNMT for SAM is very small. By contrast, AS3MT has a much higher *K*_*m*_ and therefore varies much more

A notable feature of the simulations shown in Fig. 3 is that the knockouts of PEMT, GAMT, and GNMT induce only very small changes in DNMT. The reason is easy to understand. The *K*_*m*_ of DNMT is very small, 1.4 *μ*M [36], compared to the normal concentration of SAM, 23.94 *μ*M, in the model. This means that moderate changes in SAM will hardly affect the velocity of the DNMT reaction, because the velocity curve is very flat for values of SAM considerably higher than the *K*_*m*_. One can see this effect clearly in Fig. 7, which shows hourly changes in methionine input (Panel e) due to three daily meals. GNMT (Panel c) and GAMT (Panel d) show large variations due to meals but DNMT (Panel d) shows almost no variation. This is a very simple regulatory mechanism, but it is very common. Of the 23 *K*_*m*_ values for methyltransferases listed in [1], 12 have *K*_*m*_ values ≤ 3, and 4 have *K*_*m*_ values in the range 3–10 *μ*M. If a methyltransferase has a low *K*_*m*_ value, the cell can regulate its flux simply by changing the expression level of the enzyme, independent of the fluxes of the other methyltransferases or the concentration of SAM.

### The effect of inhibition by SAH

Most methyltransferases are inhibited by SAH, the other product of the MT reaction. Of course, product inhibition is ubiquitous in cell metabolism and this simple mechanism is rightly regarded as a way of preventing the over-accumulation of product. However, the graph of PEMT (Panel d) in Fig. 7 suggests a different role for inhibition by SAH. Notice that GNMT and GAMT fluctuate up and down considerably with methionine input, while PEMT does not. All three MTs have high *K*_*m*_ values so the discussion in Section The importance of small *K*_*m*_ values is not relevant. The reason that PEMT fluctuates little is the inhibition by SAH. As indicated by Panels a and b, the SAH concentration fluctuates up and down with the SAM concentration. For PEMT, the increase in SAM (which will increase flux) is almost exactly compensated by the increase in SAH (which will decrease flux). But why doesn’t this happen for GAMT? The reason is that the *K*_*i*_ of PEMT for SAH is quite small (3.8 *μ*M) so the inhibition by SAH is quite powerful, while the *K*_*i*_ of GAMT for SAH is quite large (16 *μ*M) so the inhibition by SAH is quite weak. Thus, evolution may have tuned the *K*_*m*_ and the *K*_*i*_ values of certain methyltransferases for which it was important to buffer the flux against changes in methionine input due to meals.

### The importance of substrate availability

In this paper we have been studying the competition between different methyltransferase reactions for the methyl groups provided by SAM. Each of the methyltransferase reactions has a co-substrate to which the methyl group is given, for example phosphotidylethanolamine for the PEMT reaction or guanadinoacetate (*gaa*) for the GAMT reaction. In our simulations in Section Stabilization of methylation reactions by the long-range interactions we looked at the effects of setting one of the methylation fluxes to zero. This could happen in two ways. The expression level of the enzyme could go to zero or the concentration of the co-substrate could become zero. In general, each methylation flux can be regulated by adjusting expression level and/or substrate level. Physiological systems typically have many layers of control mechanisms and the long-range interactions discussed here are just one set of such control mechanisms in liver metabolism. Each particular methyltransferase reaction will be regulated by other mechanisms also, some of which occur in other organs. This is nicely illustrated by the GAMT reaction.

Creatine is produced by a two step process. In the first step the enzyme L-arginine:glycine amidinotransferase (AGAT) makes *gaa* in the kidney from arginine and glycine. *gaa* is exported into the plasma and is taken up by the liver where it receives a methyl group from SAM and becomes creatine via the GAMT reaction. The creatine is exported from the liver and is taken up by muscle tissue. Normally mammals ingest about 50 % of the creatine they need and synthesize the other half through these reactions [46, 47]. Thus, one would expect that creatine synthesis in the liver would be sensitive to the amount of creatine in the diet, and indeed it is [46, 47]. High plasma concentrations in the plasma inhibit AGAT in the kidney so that less *gaa* is sent to the liver and thus less creatine is produced by the GAMT reaction. The second set of bar graphs in Fig. 3a shows that if *gaa* is set to zero (for example, after a high creatine meal) and therefore the GAMT flux becomes zero, then the other MT reactions change modestly, except for GNMT that changes a lot. See the discussion in Section Stabilization of methylation reactions by the long-range interactions.

### Arsenic in Bangladesh

In this section we discuss why a full understanding of the regulation of competing methyltransferases is important for human health effects and interventions. Arsenic in drinking water is a major health hazard to millions of people in South and East Asia and in other parts of the world [48, 49]. Long term arsenic exposure has been linked to cancer, heart disease, neuropathies and neurological sequelae, and to deficits in intelligence in children [50, 51]. Arsenic in water is normally ingested primarily as trivalent inorganic arsenic (iAs), which then undergoes hepatic methylation to methylarsonic acid (MMAs) and a second methylation to dimethylarsinic acid (DMAs) by the enzyme AS3MT.

This is considered a detoxification pathway because DMAs is rapidly exported from the liver to the blood and excreted in urine. Two of us (MVG and MNH) conduct studies whose purpose is to determine which nutritional supplements would increase the rate of the AS3MT reaction and thus lower the arsenic body burden in individuals in Bangladesh. It is known both from experimentation [52] and from modeling (Panel a of Fig. 6) that an increase in folate status increases the concentration of SAM in hepatic cells. Thus one might predict that increasing folate status would increase the rate of methylation of iAs; results from a randomized clinical trial of folate supplementation to folate-deficient adults in Bangladesh show this to be true [53, 54]. Because iAs and its methylated metabolites, MMAs and DMAs, are not measured in the livers of human subjects but in blood and in urine, it is important to have a whole body mathematical model that connects arsenic metabolism in the liver to the blood and urine concentrations of iAs, MMAs, and DMAs. We created such a model in [31]. The model predictions matched the observation in the Bangladesh trials that folate supplementation of folate-deficient individuals reduced blood arsenic by 14 %. With the model, we were also able to predict that body stores of arsenic (which couldn’t be measured) would be reduced by roughly 26 %.

This led naturally to the question of whether supplementation with other nutrients, besides folate, would improve these results. The goal is to raise SAM concentrations and thus to increase the availability of methyl groups for AS3MT. A natural candidate is creatine supplementation, which has been shown to downregulate creatine biosynthesis through repression of arginine:glycine amidinotransferase (AGAT) in the kidney, which, in turn, lowers the concentration of guanidinoacetate (GAA), the substrate for GAMT in the liver [50, 51]. And this brings us immediately to the topic of the investigation in this paper, the competition between methyltransferases. If GAMT flux goes down, how much will SAM go up and how much will that increase the flux through AS3MT? We recently conducted some (unpublished) experiments with our mathematical models that suggested that creatine supplementation (in addition to folate supplementation) would lower blood arsenic an additional 8.5 %. The results in this paper help us to understand why the predicted change was modest. The long-range interactions buffer the flux through other methyltransferases when one methyl-transferase is downregulated. In a recent controlled trial in Bangladesh, 622 participants were randomized to receive 400 *μ*g folic acid, 800 *μ*g folic acid, 3 g creatine, 3 g creatine + 400 *μ*g folic acid, or placebo daily [55]. The 622 participants were comprised of both folate sufficient and deficient individuals. All participants received a water filter that removed arsenic at the start of the study. The results show that 800 *μ*g folic acid was the only treatment to lower total blood arsenic to a significantly greater extent than placebo. However, 3 g creatine+400 *μ*g folic acid did lower blood arsenic to a greater extent (14 %) than 400 *μ*g folic acid alone (3.7 %) although this finding did not reach statistical significance (*p*=0.08) [55]. Furthermore, 3 g creatine + 400 *μ*g folic acid led to decreases in GAA that were correlated with decreases in homocysteine (manuscript in preparation). Treatment effects on As methylation patterns are presently being analyzed. In general, these findings are consistent with the model predictions. Thus, to improve the outcomes, one needs to break one or more of the regulatory mechanisms. A possible way to do this is to give very high levels of folate supplementation along with the creatine supplementation. If one can raise liver folate appreciably, one could shut down the GNMT pathway because of the binding of 5mTHF to GNMT, and then creatine supplementation should have a larger effect. The difficulty is that it is easy to raise plasma folate a lot but it is not known whether this causes liver folate and liver SAM to rise a lot in folate sufficient individuals.

Liver cells are not static but are constantly changing dynamic systems because the cells must continue to operate in the face of dramatically changing inputs due to meals. It is not surprising that liver cells have evolved many complicated and ingeneous mechanisms for accomplishing the homeostasis of important reactions like the methyltransferase reactions. The purpose of our discussion of AS3MT is to illustrate that a thorough understanding of how competing methyltransferases are regulated is necessary for designing successful interventions.

## Conclusions

There are more than 150 methyltransferase reactions in which a methyl group is transferred from SAM to another substrate. These reactions are important steps in a wide variety of biological mechanisms such as the biosynthesis of a large variety of useful compounds, methylating the cytosines on DNA, and catabolizing neurotransmitters. In any given cell typically only a certain subset of the MTs are expressed depending on cell type, its function, and the tasks that the cell is currently doing. The primary mechanism used by the cell for regulating the MTs is to upregulate and downregulate the expression level of the genes that code for the MTs. However, since all the MTs use the same substrate, SAM, up and down regulation of a particular MT will affect the flux in all MT reactions, i.e. it is hard for the cell to regulate each of MT fluxes independently. It is not surprising that cells have developed a number of different mechanisms that help them meet this challenge. In addition, cells must cope with large changes in amino acid input (i.e. methionine) that happen on a shorter time scale than gene regulation.

In this paper, we have used a mathematical model to investigate the mechanisms that liver cells use to manage the non-independence of the MT fluxes.

- We have found that long-range allosteric interactions in which substrates in one part of the network affect enzyme activity in distant parts of the network play an important role:
  - The long-range interactions ensure that when one MT is greatly upregulated or downregulated the GNMT pathway changes a lot so that the fluxes in the other MT pathways change only modestly (Fig. 3). SAM changes much more in the presence of MT up and down regulation without the long-range interactions (Fig. 4).
  - The long-range interactions act to conserve total mass in the methionine cycle. SAM changes linearly and slowly as methionine input changes (Fig. 5).
  - The long-range interactions stabilize total methylation against folate deficiency (Fig. 6b).
- We have provided computational evidence that GNMT that is bound to one 5mTHF molecule retains substantial activity. This remains to be confirmed experimentally.
- We explained why the small *K*_*m*_ values of many MTs for SAM makes them independent of the fluxes through other MT reactions.
- We explained why the small *K*_*i*_ value of SAH for PEMT stabilizes PEMT flux.

And finally, we discussed why a full understanding of the regulation of MT fluxes is important for human health interventions to ameliorate arsenic toxicity in Bangladesh.

## Additional file

Additional file 1 **Supplementary material.** (PDF 155 KB)

## Abbreviations

MT: Methyltransferase
Met: Methionine
SAM: S-adenosylmethionine
SAH: S-adenosylhomocysteine
Hcy: Homocysteine
5mTHF: 5-methyltetrahydrofolate
THF: Tetrahydrofolate
10fTHF: 10-formyltetrahydrofolate
DHF: Dihydrofolate
CH2-THF: 5,10-methylenetrahydrofolate
CH=THF: 5,10-methenyltetrahydrofolate
AICAR(T): Aminoimidazolecarboxamide ribonucleotide (transferase)
FTD: 10-formyltetrahydrofolate dehydrogenase
FTS: 10-formyltetrahydrofolate synthase
MTCH: 5,10-methylenetetrahydrofolate cyclohydrolase
MTD: 5,10-methylenetetrahydrofolate dehydrogenase
MTHFR: 5,10-methylenetetrahydrofolate reductase
TS: Thymidylate synthase
SHMT: Serine hydroxymethyltransferase
PGT: Phosphoribosyl glycinamidetransformalase
DHFR: Dihydrofolate reductase
NE: Nonenzymatic interconversion of THF and 5,10-CH2-THF
MAT-I: Methionine adenosyl transferase I
MAT-III: Methionine adenosyl transferase III
GNMT: Glycine N-methyltransferase
AS3MT: Arsenic methyltransferase
PEMT: Phosphotidylethanolamine methyltransferase
GAMT: Gunadino-acetate methyltransferase
DNMT: DNA-methyltransferase
SAHH: S-adenosylhomocysteine hydrolase
CBS: Cystathionine β-synthase
MS: Methionine synthase
BHMT: Betaine-homocysteine methyltransferase
*V*_*max*_: The maximum velocity of a reaction
*K*_*m*_: The Michaelis constant for an enzyme
*K*_*i*_: The inhibition constant for an enzyme.
