# Supplementary material for: Mathematical analysis of the regulation of competing methyltransferases
Source: BMC Syst Biol. 2015 Oct 14;9:69. doi: 10.1186/s12918-015-0215-6 (PMC4606511; doi:10.1186/s12918-015-0215-6)
Supplement: Supplementary file 1 — Supplementary material. (PDF 155 KB) [file 12918_2015_215_MOESM1_ESM.pdf]

Supplementary Material

for

Mathematical analysis of the regulation of competing methyltransferases

M. Reed[1], M. Gamble[2], M. Hall[2], H. F. Nijhout[3]

[1] Department of Mathematics, Duke University
[2] Mailman School of Public Health, Columbia University
[3] Department of Biology, Duke University

Corresponding author: Michael C. Reed, Department of Mathematics, Duke University, Durham, NC 27708. email: reed@math.duke.edu, phone: 919-660-2808, FAX: 919-660-2821.

Keywords: mathematical model, methyltransferase, regulation, folate, arsenic

In these supplementary materials we give the full details of the mathematical model. Figure 1 shows a schematic diagram of the biochemical reactions in the model. Full substrate and enzyme names are given in the legend.

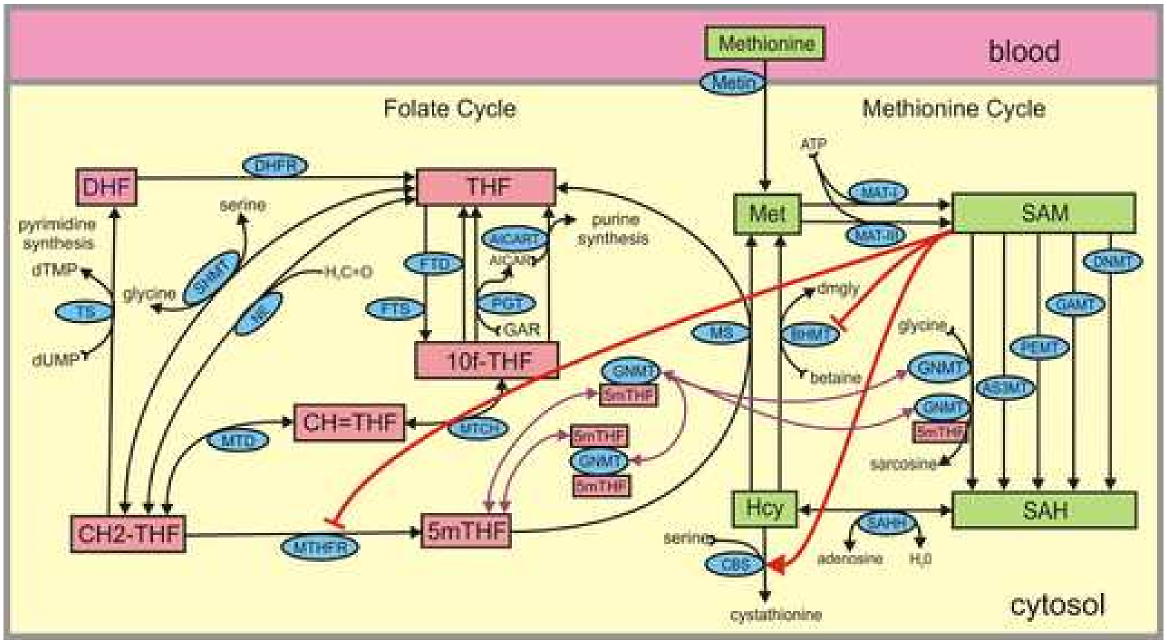

Figure S1. Folate and methionine metabolism with competing methyltransferases. Substrates are indicated by rectangular boxes, green in the methionine cycle and red in the folate cycle, except for GNMT which is both an enzyme and a substrate since it can bind to two molecules of 5mTHF. Each arrow represents a biochemical reaction and the blue ellipse on the arrow contains the acronym of the enzyme that catalyzes the reaction. Substrate abbreviations: Met, methionine; SAM, S-adenosylmethionine; SAH, S-adenosylhomocysteine; Hcy, homocysteine; 5mTHF, 5-methyltetrahydrofolate; THF, tetrahydrofolate; 10fTHF, 10-formyltetrahydrofolate; DHF, dihydrofolate; CH2-THF, 5,10-methylenetrahydrofolate; CH=THF, 5,10-methenyltetrahydrofolate. Enzyme abbreviations: AICAR(T), aminoimidazolecarboxamide ribonucleotide (transferase); FTD, 10-formyltetrahydrofolate dehydrogenase; FTS, 10-formyltetrahydrofolate synthase; MTCH, 5,10-methylenetetrahydrofolate cyclohydrolase; MTD, 5,10-methylenetetrahydrofolate dehydrogenase; MTHFR, 5,10-methylenetetrahydrofolate reductase; TS, thymidylate synthase; SHMT, serine hydroxymethyltransferase; PGT, phosphoribosyl glycinamidetransformalase; DHFR, dihydrofolate reductase; NE, nonenzymatic interconversion of THF and 5,10-CH2-THF; MAT-I,methionine adenosyl transferase I; MAT-III, methionine adenosyl transferase III; GNMT, glycine N-methyltransferase; AS3MT, arsenic methyltransferase; PEMT, phosphotidylethanolamine methyltransferase; GAMT, gunadino-acetate methyltransferase; DNMT, DNA-methyltransferase; SAHH, S-adenosylhomocysteine hydrolase; CBS, cystathionine β-synthase; MS, methionine synthase; BHMT, betaine-homocysteine methyltransferase.

In specifying the differential equations, we use lower case letters and simple abbreviations for the substrates; these abbreviations are indicated in Table 1, below. Velocities are always indicated by V_X where the subscript X gives the name of the enzyme that catalyzes that

particular velocity. Each velocity depends, of course, on the current values of various of the substrates.

Table S1, Variable names and usual acronyms

variable	usual acronym
met	MET
sam	SAM
sah	SAH
hcy	HCY
dhf	DHF
thf	THF
fthf	10f-THF
ch	CH=THF
ch2	CH2-THF
mthf	5mTHF
gnmt	GNMT
gnmtf	GNMT-5mTHF
fgnmtf	5mTHF-GNMT-5mTHF

The 13 differential equations are simply mass balance equations that say that the rate of change of the concentration of a substrate is the sum of the velocities of the reactions that make the substrate minus the sum of the reactions that use the substrate. The differential equations follow:

$$
\begin{aligned}
\frac{d}{dt}(met) &= metin(t) + V_{\mathrm{BHMT}}(hcy, bet, sam, sah) + V_{\mathrm{MS}}(mthf, hcy) - V_{\mathrm{MATI}}(met, sam) \\
&\quad -V_{\mathrm{MATIII}}(met, sam) \\
\frac{d}{dt}(sam) &= V_{\mathrm{MATI}}(met, sam) + V_{\mathrm{MATIII}}(met, sam) - V_{\mathrm{GNMT}}(sam, sah, gly, gnmt, gnmtf) \\
&\quad -V_{\mathrm{AS3MT}}(sam, sah, ias) - V_{\mathrm{PEMT}}(sam, sah, pe) - V_{\mathrm{GAMT}}(sam, sah, gaa) - V_{\mathrm{DNMT}}(sam) \\
\frac{d}{dt}(sah) &= V_{\mathrm{GNMT}}(sam, sah, gly, gnmt, gnmtf) + V_{\mathrm{AS3MT}}(sam, sah, ias) + V_{\mathrm{PEMT}}(sam, sah, pe) \\
&\quad +V_{\mathrm{GAMT}}(sam, sah, gaa) + V_{\mathrm{DNMT}}(sam) - V_{\mathrm{SAAH}}(sah, hcy) \\
\frac{d}{dt}(hcy) &= V_{\mathrm{SAAH}}(sah, hcy) - V_{\mathrm{BHMT}}(hcy, bet, sam, sah) - V_{\mathrm{MS}}(mthf, hcy) - V_{\mathrm{CBS}}(hcy, sam, sah, ser) \\
\frac{d}{dt}(dhf) &= V_{\mathrm{TS}}(dump, ch2) - V_{\mathrm{DHFR}}(dhf, nadph) \\
\frac{d}{dt}(thf) &= V_{\mathrm{DHFR}}(dhf, nadph) + V_{\mathrm{MS}}(mthf, hcy) + V_{\mathrm{FTD}}(fthf) + V_{\mathrm{PGT}}(fthf, gar) + V_{\mathrm{AICART}}(fthf, aic) \\
&\quad -V_{\mathrm{FTS}}(thf, hcooh, fthf) - V_{\mathrm{SHMT}}(ser, thf, gly, ch2) - V_{\mathrm{NE}}(thf, hcoh, ch2) \\
\frac{d}{dt}(fthf) &= V_{\mathrm{MTCH}}(ch, fthf) + V_{\mathrm{FTS}}(thf, hcooh, fthf) - V_{\mathrm{PGT}}(fthf, gar) \\
&\quad -V_{\mathrm{AICART}}(fthf, aic) - V_{\mathrm{FTD}}(fthf) \\
\frac{d}{dt}(ch) &= V_{\mathrm{MTD}}(ch2, ch) - V_{\mathrm{MTCH}}(ch, fthf) \\
\frac{d}{dt}(ch2) &= V_{\mathrm{SHMT}}(ser, thf, gly, ch2) + V_{\mathrm{NE}}(thf, hcoh, ch2) - V_{\mathrm{TS}}(dump, ch2) \\
&\quad -V_{\mathrm{MTD}}(ch2, ch) - V_{\mathrm{MTHFR}}(ch2, nadph, sam, sah) \\
\frac{d}{dt}(mthf) &= V_{\mathrm{MTHFR}}(ch2, nadph, sam, sah) - V_{\mathrm{MS}}(mthf, hcy) + k_2(gnmtf) \\
&\quad -2k_1(mthf)(gnmt) + k_4(fgnmtf) - k_3(mthf)(gnmtf) \\
\frac{d}{dt}(gnmt) &= k_2(gnmtf) - 2k_1(mthf)(gnmt) \\
\frac{d}{dt}(gnmtf) &= -k_2(gnmtf) + 2k_1(mthf)(gnmt) - k_3(mthf)(gnmtf) + k_4(fgnmtf) \\
\frac{d}{dt}(fgnmtf) &= k_3(mthf)(gnmtf) - k_4(fgnmtf)
\end{aligned}
$$

Some of the reactions depend on the concentrations of other substrates that are not variable (in the model) and are assumed to be constant. These are give in Table S2.

Table S2. Constant concentrations (μM) in the model

abbreviation	value	name
aic	2.1	AICARP
bet	50	betaine
dump	20	DUMP
gaa	10	guanadinoacetate
gar	10	GAR
gly	1850	glycine
hcoh	500	HCOH (formaldehyde)
hcooh	900	HCOOH (formate)
ias	1	inorganic arsenic
nadph	50	NADPH
pe	10	phosphotidylethanolamine
ser	468	serine

The details of the biochemistry and the biology are in the functional forms that show how each of the velocities depends on the current values of the variables that influence it. Many reactions have Michaelis-Menten kinetics in one of the following standard forms:

$$V = \frac{V_{max}[S]}{K_m + [S]}, \qquad V = \frac{V_{max}[S_1][S_2]}{(K_{S_1} + [S_1])(K_{S_2} + [S_2])}$$

$$V = \frac{V^f_{max}[S_1][S_2]}{(K_{S_1} + [S_1])(K_{S_2} + [S_2])} - \frac{V^b_{max}[P_1][P_2]}{(K_{P_1} + [P_1])(K_{P_2} + [P_2])}$$

for unidirectional, one substrate, unidirectional, two substrates, and bidirectional, two substrates, two products, respectively. For these reactions, Table S3 lists the K_m and V_{max} values. In general, we take K_m values from the literature. V_{max} values are extremely variable because they depend on enzyme expressions levels that vary in time and therefore experimental measurements *in vivo* are difficult and unreliable. We usually adjust the V_{max} values so as to obtain the typical substrate concentration values that we find in the literature. Parameters have sometimes been chosen by comparing model outputs in various circumstances to qualitative and quantitative experimental data.

Table S3. Model kinetic parameters (time in hrs, concentration in μM)

parameter	literature	model	reference
**AICART**			
$K_{m,fthf}$	5.9-50	5.9	[1][2][3][4]
$K_{m,aicar}$	10-100	100	[1][2][4]
V_{max}		55000	
**DHFR**			
$K_{m,dhf}$	0.12-1.9	0.5	[2][4][5][6]
$K_{m,nadph}$	0.3-5.6	4.0	[2][4][5][6]
V_{max}	350-23000	2000	[2][4][5]
**FTD**			
$K_{m,fthf}$	0.9-20	20	[7][8]
V_{max}		500	
**FTS**(forward direction from thf to fthf)			
$K_{m,thf}$	0.1-600	3	[3][4]
$K_{m,hcooh}$	8-1000	43	[3][4]
V_{max}	100-468000	3900	[3][4]
**MS**			
$K_{m,mthf}$	22-34	25	[9][10]
$K_{m,thf}$	0.1-6	1	[11]
V_{max}		244	[11]
**MTCH**(forward direction from ch to fthf)			
$K_{m,ch}$	4-250	250	[2][3][4]
V_{max}	880-1380000	500000	[2][3]
$K_{m,fthf}$	20-450	100	[2][3][4]
V_{max}	10.5-1380000	20000	[2][3]
**MTD**(positive direction from ch2 to ch)			
$K_{m,ch2}$	2-5	2	[3][4]
V_{max}	520-594000	80000	[5][3][4]
$K_{m,ch}$	1-10	10	[3][12]
V_{max}	594000	600000	[3]
**PGT**			

$K_{m,fthf}$	4.9-58	4.9	[4][2][13][14]
$K_{m,gar}$	520	520	[4][2][13][14]
V_{max}	6600-16200	24300	[4][2][13][14]
**SAHH**			
$K_{m,sah}$	0.75-15.2	6.5	[15][16][17][18]
V_{max}		320	
$K_{m,hcy}$	56.6-200	150	[16][17][19]
**SHMT**(positive direction is from thf to ch2)			
$K_{m,ser}$	350-1300	600	[2][3][4][20]
$K_{m,thf}$	45-300	50	[2][3][4][21]
V_{max}	500-162000	5200	[2][3][21]
$K_{m,gly}$	3000-10000	10000	[2][3][4][20][5]
$K_{m,ch2}$	3000-10000	3200	[2][3][5][21]
V_{max}	12600-120000000	15000000	[2][3][5]

Now we discuss in detail the methylation reactions, the more difficult modeling issues, and reactions with non-standard kinetics.

AS3MT. Inorganic arsenic is metabolized in two methylation steps catalyzed by AS3MT. The first step uses utilizes a methyl group from SAM and is followed by a reduction step to produce methylarsonic acid (MMA). The second step uses utilizes a methyl group from SAM and is followed by a reduction step to produce dimethyarsinic acid (DMA), which is readily exported from the liver and cleared in the urine. We have recently studied the biochemistry of these methylation steps that are quite complicated [22]. For, example the first step shows substrate inhibition by inorganic arsenic and product inhibition by MMA and glutathione (GSH) both sequesters the arsenic compounds and activates AS3MT. In our study here, we are mainly interested in studying the availability of methyl groups from SAM, so we take the arsenic concentrations and the GSH concentration to be constant, and model just the first methylation step. SAM shows substrate inhibition for AS3MT [23], but the effect is small and occurs only at very high SAM concentrations, so we ignore it. Thus, the velocity of methylation is taken to be:

$$V_{\mathrm{AS3MT}}(sam, sah, ias) \ = \ \frac{V_{max}(sam)}{(K_m(1+\frac{sah}{K_i})+sam)} \cdot \frac{iAs}{4.6+iAs}.$$

We take the K_m of AS3MT for SAM to be 50μM as determined in [24] and the K_m for iAs to be 4.6μM [25]. It is known that SAH inhibits AS3MT [26, 27], but the nature of the inhibition and the K_i are not known. We'll assume the inhibition is competitive and take $K_i = 10\mu$M, which is typical of other methyltransferases. A high, but realistic arsenic load

is 1 μM in liver [28] and a typical flux would be the order of magnitude of 1 μM/hr. So, we choose $V_{max} = 28\mu$/hr, which accomplishes this given that a typical SAM concentration is 24 μM.

BHMT. The kinetics of BHMT are Michaelis-Menten with the parameters $K_{m,hcy} = 12, K_{m,bet} = 100$, and $V_{max} = 502$ [29][30]. The form of the inhibition of BHMT by SAM and SAH was derived by non-linear regression on the data of [31] and scaled so that it equals 1 at the normal methionine input of 50μM/hr.

$$V_{\mathrm{BHMT}}(hcy, bet, sam, sah) \;=\; \frac{V_{max}(hcy)(bet)}{(K_{m,hcy}+hcy)(K_{m,bet}+bet)} \cdot e^{-.0021(sam+sah)}e^{.0021(28)}$$

Binding of 5mTHF to GNMT. In a series of papers, Wagner, Luka, and colleagues have studied the inhibitory effect of 5mTHF on the activity of GNMT [32, 23, 33, 34, 35]. GNMT has two binding sites for 5mTHF, so we assume the simple reversible reactions:

$$5mTHF \;+\; GNMT \;\rightleftarrows\; 5mTHF\text{-}GNMT$$

$$5mTHF\text{-}GNMT \;+\; 5mTHF \;\rightleftarrows\; 5mTHF\text{-}GNMT\text{-}5mTHF$$

with forward and backward rate constants, k_1 and k_2, for the first reaction and k_3 and k_4, for the second reaction. We choose the rate constants $k_1 = 50, k_2 = 1, k_3 = 1, k_4 = 1.6$ so that the K_D values are those found in Table 2 of [33].

CBS The kinetics of CBS are standard Michaelis-Menten with $K_{m,1} = 1000$ for hcy taken from [36] and $K_{m,2} = 2000$ for ser taken from [37], with $V_{max} = 117{,}000$. The form of the activation of CBS by sam and sah was derived by non-linear regression on the data in [38] and [39] and scaled so that it equals 1 when the external methionine concentration is 30 μM.

$$V_{\mathrm{CBS}} \;=\; \left(\frac{V_{max}(hcy)(ser)}{(K_{m,1}+hcy)(K_{m,2}+ser)}\right)\left(\frac{(1.2)(sam+sah)^2}{((30)^2+(sam+sah)^2}\right)\left(\frac{(1.2)(28)^2}{((30)^2+(28)^2}\right)^{-1}.$$

DNMT. The velocity of the DNMT reaction is given by

$$V_{\mathrm{DNMT}}(sam, sah) \;=\; \frac{V_{max}(sam)}{K_m(1+\frac{(sah)}{K_i})+(sam)}.$$

The inhibition by SAH is competitive [40]. We choose $K_m = 1.4\mu$M for SAM and $K_i = 1.4\mu$M for SAH as indicated in [41]. The reaction depends on the cytosines available, but since we take their concentration to be constant we fold that dependence into the V_{max}. The value $V_{max} = 12.5\mu$M/hr was chosen so that the flux of the DNMT reaction is normally (when the cell is not dividing) much less than the fluxes of GNMT, PEMT, and GAMT.

GAMT. The velocity of the GAMT reaction is given by

$$V_{\text{GAMT}}(sam, sah, gaa) = \frac{V_{max}(sam)(gaa)}{(K_m(1+\frac{sah}{K_i}) + sam)(K_m + gaa)}.$$

The inhibition by SAH is competitive [42, 43]. We choose $K_m = 49\mu$M for SAM and $K_i = 16\mu$M for SAH as indicated in [44] and take $K_m = 13.3\mu$M for *gaa* as in [45]. The value $V_{max} = 210\mu$M/hr was chosen so that the flux of the GAMT reaction is comparable to the fluxes of the GNMT and PEMT reactions, the two other methyl transferases that carry much of the methylation flux.

GNMT. The kinetics of GNMT for SAM are cooperative and we take the Hill coefficient to be $n = 2$ as suggested in [23] and we use $K_m = 100\mu$M as indicated in [44]. The inhibition by SAH is competitive [46] and has $K_i = 35\mu$M [44]. The reaction has glycine as a substrate and we take $K_m = 12.2\mu$M of GNMT for glycine as found in [**?**]. Thus,

$$V_{\text{GNMT}}(sam, sah, gnmt, gnmtf) = \frac{V_{max}(sam)^2}{(K_m(1+\frac{sah}{K_i}))^2 + (sam)^2])} \frac{gly}{(K_m + gly)}.$$

where

$$V_{max} = (4026)(gnmt + (.5)(gnmtf)).$$

This formula for V_{max} resulted from our *in silico* experiments described under Results 3.1. The concentration of free GNMT, gnmt, is a variable in our model. GNMT can be bound by one or two molecules of 5mTHF. Our simulations and the data in [33] suggest strongly that once bound GNMT, namely gnmtf, retains 50% of it's activity. The factor 4026 is chosen so that GNMT has a normal reaction velocity comparable to the reaction velocities of PEMT and GAMT, the two other methyl transferases that carry much of the methylation flux.

MAT-I. The MAT-I kinetics are from [47], Table 1, and we take $V_{max} = 260$ and $K_m = 41$. The inhibition by SAM was derived by non-linear regression on the data from [47], Figure 5.

$$V_{\text{MAT-I}} = \left(\frac{V_{max}(met)}{K_m + met)}\right)(0.23 + (0.8)e^{-(0.0026)(sam)})$$

MAT-III. The methionine dependence of the MAT-III kinetics is from [48], Figure 5, fitted to a Hill equation with $V_{max} = 220$, $K_m = 300$. The activation by SAM is from [47], Figure 5, fitted to a Hill equation with K_a= 360. We model the activation of MATIII by SAM by effectively changing the V_{max}, but, in fact, SAM lowers the K_m for methionine; for a detailed discussion, see [49].

$$V_{\text{MAT-III}} = \left(\frac{V_{max}(met)^{1.21}}{K_m + (met)^{1.21}}\right)\left(1 + \frac{(7.2)(sam)^2}{K_a^2 + (sam)^2}\right)$$

MTHFR. The first factor in the formula for the MTHFR reaction velocity

$$V_{\text{MTHFR}} = \left(\frac{V_{max}(met)(nadph)}{(K_{m,1} + ch2)(K_{m,2} + nadph)}\right)\left(3. * \frac{10}{10 + (sam - sah)}\right)$$

is standard Michaelis-Menten with $K_{m,1} = 50$, $K_{m,2} = 16$, and $V_{max} = 5300$ taken from [50][51][52]. The inhibition of MTHFR by SAM, the second factor, was derived by non-linear regression on the data of [53][54] and has the form 10/(10+ sam). In addition, SAH competes with sam for binding to the regulatory domain of MTHFR. It neither activates nor inhibits the enzyme [54] but prevents inhibition by sam; thus, we take our inhibitory factor to be:

$$\frac{10}{10 + (sam - sah)}.$$

The factor 3 scales the inhibition so that it has value 1 when the external methionine input is 50 μM/hr.

NE. The kinetics of the non-enzymatic reversible reaction between thf and ch2 are taken to be mass action,

$$V_{\text{NE}} = k_1(thf)(hcho) - k_2(ch2),$$

with rate constants are $k_1 = 0.03$, and $k_2 = 22$. *hcho* represents formaldehyde.

PEMT. The velocity of the PEMT reaction is given by

$$V_{\text{PEMT}}(sam, sam, pe) = \frac{V_{max}(sam)}{(K_m + (sam))(1 + \frac{sah}{K_i})} \frac{pe}{(K_m + pe)}.$$

The inhibition by SAH is non-competitive [55]. We choose $K_m = 18.2\mu$M for SAM and $K_i = 3.8\mu$M for SAH as indicated in [44]. The reaction depends on *pe* (phophatidylethanolamine) and we take $K_m = 5000\mu$M of PEMT for *pe* as found in [55].The value $V_{max} = 49100\mu$M/hr was chosen so that the flux of the PEMT reaction is comparable to the fluxes of the GNMT and GAMT reactions, the two other methyl transferases that carry much of the methylation flux.

Acknowledgements This research was partially supported by NSF EF-1038593 (HFN,MR), and NIH grants R01 ES019876 (D. Thomas), RO1 CA133595, RO1 ES017875, P42 ES10349 (MGV) and K99ES018890 (MNH).

Conflict of Interest Statement. The authors declare that they have no conflicts of interest.

References

[1] Rayl EA, Moroson BA, Beardsley GP: **The human purH gene product, 5-aminoimodazole-4-carboxamide ribonucleotide formyltransferase/IMP cyclohydrolase. Cloning, sequence, expression, purification, kinetic analysis, and domain mapping**. *J. Biol. Chem.* 1996, **271**:2225–2233.

[2] Seither R, Trent DF, Mickulecky DC, Rape TJ, Goldman ID: **Folate-pool interconversions and inhibition of biosynthetic processes after exposure of L1210 leukemia cells to antifolates**. *J. Biol. Chem.* 1989, **264**:17016–17023.

[3] Strong WB, Tendler SJ, Seither RL, Goldman ID: **Purification and Properties of Serine Hydroxymethyltransferase C1-Tetrahydrofolate Synthase from L1210 Cells**. *J. Biol. Chem.* 1990, **265**:12149–12155.

[4] Vorontzov IN, Greshilov MM, Belousova AK, Gerasimova GK: **Mathematical description and investigation of the principles of functioning of the folic acid cycle**. *Biokhimiya* 1980, **45**:83–97.

[5] Jackson RC, Harrup KR: **Studies with a mathematical model of folate metabolism.** *Arch. Biochem. Biophys.* 1973, **158**:827–841.

[6] Blake RL: **Eukaryotic dihydrofolate reductase**. *Adv. Enzymol.* 1995, **60**:23–.

[7] Schirch D, Villar E, Mara B, Barra D, Schrich V: **Domain structure and function of 10-formyltetrahydrofolate dehydrogenase**. *J. Biol. Chem.* 1994, **269**:24728–24735.

[8] Kim DW, Huang T, Schirch D, Schrich V: **Properties of Tetrahydropteroylpentaglutamate bound to 10-formyltetrahydrofolate dehydrogenase**. *Biochem.* 1996, **35**:15772–15783.

[9] Finkelstein JD, Martin JJ: **Methionine metabolism is mammals: Adaptation to methionine excess**. *J. Biol. Chem.* 1986, **261**:1582–1587.

[10] Banerjee R, Frasca V, Ballou D, Matthews R: **Participation of Cob(I)alamin in the reaction catalyzed by methionine synthase from Escherichia coli: a steady state and rapid reaction kinetic analysis,**. *Biochem.* 1990, **29**:11101–11109.

[11] Banerjee R, Chen Z, Gulati S: **Methionine synthase from pig liver**. *Mewth. Enzymol.* 1997, **281**:189–197.

[12] Wagner C: *Folate in Health and Disease*, New York: Marcel Dekker 1995 chap. Biochemical role of folate in cellular metabolism, :23–42.

[13] Caperelli CA: **Mammalian glycinamide ribonucleotide transformylase: purification and some properties**. *Biochemistry* 1985, **24**:1316–1320.

[14] Caperelli CA: **Mammalian glycinamide ribonucleotide transformylase. Kinetic mechanism and associated de novo purine biosynthetic activities**. *J. Biol. Chem.* 1989, **264**:5053–5057.

[15] Doskeland SO, Ueland PM: **Comparison of some physicochemical and kinetic properties of S-adenosylhomocysteine hydrolase from bovine liver, bovine adrenal cortex and mouse liver**. *Biochim Biophys Acta* 1982, **708**:185–193.

[16] Fujioka M, Takata Y: **S-Adenosylhomocysteine Hydrolase from rat liver: Purification and some properties**. *J. Biol. Chem.* 1981, **256**:1631–1635.

[17] Hershfield M, Aiyar VN, Premakumar R, Small WC: **S-Adenosylhomocysteine hydrolase from human placenta**. *J. Biochem.* 1985, **230**:43–52.

[18] Klor D, Kurz J, Fuch S, Faust B, Osswald H: **S-adenostlhomocysteine-hydrolase from bovine kidney: enzymatic and binding properties**. *Kid. Blood Press. Res.* 1996, **19**:100–108.

[19] Gomi T, Takata Y, Date T, Motoji F, Akasamit RR, Backlund P, Cantoni G: **Site-directed mutagenesis of rat liver S-Adenosylhomocysteine**. *J. Biol. Chem.* 1990, **265**:16101–16107.

[20] Schirch V, Hopkins S, Villar E, Angelaccio S: **Serine hydroxymethyltransferase from Escherichia coli: purification and properties**. *J. Bacteriol.* 1985, **163**:1–7.

[21] Schirch V: **Purification and properties of folate-dependent enzymes from rabbit liver**. *Meth. Enzymol.* 1997, **281**:146–161.

[22] Lawley SD, Yun J, Gamble MV, Hall MN, Reed MC, Nijhout HF: **Mathematical modeling of the effects of glutathione on arsenic methylation**. *Theor. Biol. Med. Model.* 2014, **11**:20–.

[23] Yeo EJ, Briggs WT, Wagner C: **Inhibition of Glycine N-Methyltransferase by 5-Methyltetrahydrofolate Pentaglutamate**. *The Journal of Biological Chemistry* 1999, **274**(53):37559–37564.

[24] Song X, Geng Z, Li X, Hu X, Bian N, Zhang X, Wang Z: **New insights into the mechanism of arsenite methylation with the recombinant human arsenic (3) methyltransferase (hAS3MT)**. *Biochimie* 2010, **92**:1397–1406.

[25] Wood TC, Salavigionne OE, Mukherjee B, Wang L, Klumpp AF, Thomae BA, Eckloff BW, Schaid DJ, Wieben ED, Weinshilboum RM: **Human Arsenic Methyltransferase (AS3MT) Pharmacogenetics**. *The Journal of Biological Chemistry* 2006, **281**(11):7364–7373.

[26] Styblo M, Delnomdedieu M, Thomas DJ: **Mono- and dimethylation of arsenic in rat liver cytosol in vitro**. *Chemico-biological interactions* 1996, **99**:147–164.

[27] DeKimpe J, Cornelius R, Vanderholder R: **In vitro methylation of arsenite by rabbit liver cytosol: effect of metal ions, metal chelating agents, methyltransferase inhibitors and uremic toxins**. *Drug. Chem. Toxicol.* 1999, **22**:613–628.

[28] Lawley SD, Cinderella M, Hall MN, Gamble MV, Nijhiout HF, Reed MC: **Mathematical model insights into arsenic metabolism**. *Theor. Biol. Med. Model.* 2011, **8**:31–.

[29] Finkelstein JD, Harris BJ, Kyle WE: **Methionine metabolism in mam- mals: kinetic study of betaine-homocysteine methyltransferase**. *Arch. Biochem. Biophys.* 1972, **153**:320–324.

[30] Skiba WE, Taylor MP, Wells MS, Mangum JH, Awad WM: **Human hepatic methionine biosynthesis. Purification and characterization of be- taine:homocysteine S-methyltransferase**. *J. Biol. Chem.* 1982, **257**:14944–14948.

[31] Finkelstein JD, Martin JJ: **Methionine metabolism is mammals. Distribution of homocysteine between competing pathways**. *J. Biol. Chem.* 1984, **259**:9508–9513.

[32] Wagner C, Briggs WT, Cook RJ: **Inhibition of Glycine N-Methyltransferase Acitivty by Folate Derivatives: Implications for Regulation of Methyl Group Metabolism**. *Biochemical and Biophysical Research Communications* 1985, **127**:746–752.

[33] Luka Z, Loukchevitch LV, Wagner C: **Acetylation of N-terminal valine of glycine N-methyltransferase affects enzyme inhibition by folate**. *Biochem. Biophys. Acta* 2008, **1794**(9):1342–1346.

[34] Luka Z, Mudd SH, Wagner C: **Glycine N-Methyltransferase and Regulation of S-Adenosylmethionine Levels**. *J. Biol. Chem.* 2009, **284**:22507–22511.

[35] Luka Z, Pakhomova S, loukachevitch LV, newcomer ME, Wagner C: **Differences in folate–protein interactions result in differing inhibition of native rat liver and recombinant glycine N-methyltransferase by 5-methyltetrahydrofolate**. *Biochem. Biophys. Acta* 2012, **1824**:286–291.

[36] Finkelstein JD: *Homocysteine Metabolism is Health and Disease*, Cambridge University Press 2001 chap. Regulation of homocysteine metabolism.

[37] Taoka S, Ohja S, Shan X, Kruger WD, Banerjee R: **Evidence for heme-mediated redox regulation of human cystathionine beta-synthase activity**. *J. Biol. Chem.* 1998, **273**:25179–25184.

[38] Janosik M, Kery V, Gaustadnes M, Maclean KN, Kraus JP: **Regulation of human cystathionine beta-synthase by S-adenosyl-L-methionine: Evidence for two catalytically active conformations involving an autoinhibitory domain in the C-terminal region.** *Biochemistry* 2001, **40**:10625–10633.

[39] Kluijtmans LAJ, Boers GHJ, Stevens EMB, Renier WO, Kraus JP, Trijbels FJM, Heuvel LPWJ, Blom HJ: **Defective cystathionine beta-synthase regulation by S-adenosylmethionine in a partially pyridoxine responsive homocystinuria patient**. *J. Clin Invest.* 1996, **98**:285–289.

[40] Simon D, Grunert F, v Acken U, Doring HP, Kroger H: **DNA-methylase from regenerating rat liver: purification and characterisation**. *Nuceic Acids Res.* 1978, **5**(6):2153–2167.

[41] Flynn J, Reich N: **Murine DNA (cytosine-5-)-methyltransferase: Steady-state and substrate trapping analyses of the kinetic mechanism**. *Biochemistry* 1998, **37**:15162–15169.

[42] Im YS, Chiang PK, Cantoni GL: **Guanidoacetate methyltransferase. Purification and molecular properties.** *J. Biol. Chem.* 1979, **254**:11047–11050.

[43] Takata Y, Fujioka M: **Identification of a tyrosine residue in rat guanidinoacetate methyltransferase that is photolabeled with S-Adenosyl-L-methionine**. *Biochemistry* 1992, **31**:4369–4374.

[44] Clarke S, Banfield K: *Homocysteine in Health and Disease (Ed. R. Carmel and D. W. Jacobsen)*, Cambridge University Press. 7 2001 chap. S-Adenosylmethionine-dependent methyltransferases.

[45] daSilva RP, Nissim I, Brosnan ME, Brosnan JT: **Creatine synthesis: hepatic metabolism of guanidinoacetate and creatine in the rat in vitro and in vivo.** *Am. J. Physiol. Endocrinol. metab.* 2009, **296**:E256–E261.

[46] Heady JE, Kerr SJ: **Purification and Characterization of Glycine N-Methyltransferase**. *The Journal of Biological Chemistry* 1973, **248**:69–72.

[47] Sullivan DM, Hoffman JL: **XXX**. *Biochem.* 1983, **22**:1636–1641.

[48] SanchezdelPino MM, Corrales FJ, Mato JM: **XXXXXX**. *J. Biol. Chem.* 2000, **275**:23476–23482.

[49] Korendyaseva TK, Kuvatov DN, Volkov VA, Martinov MV, Vivitsky VM, banerjee R, Ataullakhanov FI: **An Allosteric Mechanism for Switching between Parallel Tracks in Mammalian Sulfur Metabolism**. *PLOS Computational Biology* 2008, **4**:1–10.

[50] Matthews RG: **methylenetetrahydrofolate reductase from pig liver**. *Meth. Enzymol.* 1986, **122**:372–381.

[51] Green JM, MacKensie RE, Matthews RG: **Substrate flux through methylenetetrahydrofolate dehydrogenase: Predicted effects of the concentrtion of methylenetetrahydrofolate on its partitioning into pathways leading to nucleotide biosynthesis or methionine regeneration**. *Biochem.* 1988, **27**:8014–8022.

[52] Daubner SC, Matthews RG: **Purification and properties of methylenete- trahydrofolate reductase from pig liver**. *J. Biol. Chem.* 1982, **257**:140–145.

[53] Jencks DA, Matthews RG: **Allosteric inhibition of methylenetetrahydrofolate reductase by adenosylmethionine. Effects of adenosylmethionine and NADPH on the equilibrium between active and inactive forms of the enzyme and on the kinetics of approach to equilibrium**. *J. Biol. Chem.* 1987, **262**:2485–2493.

[54] Yamada K, Chen Z, Rozen R, Matthews RG: **Effects of common polymorphisms on the properties of recombinant human methylenetetrahydrofolate reductase**. *PNAS* 2001, **98**:14853–14858.

[55] Vance DE, Ridgway ND: **The methylation of phosphatidylethanolamine**. *Prog. Lip. Res.* 1988, **27**:61–79.
